# Effect of Novel, School-Based High-Intensity Interval Training (HIT) on Cardiometabolic Health in Adolescents: Project FFAB (Fun Fast Activity Blasts) - An Exploratory Controlled Before-And-After Trial

**DOI:** 10.1371/journal.pone.0159116

**Published:** 2016-08-03

**Authors:** Kathryn L. Weston, Liane B. Azevedo, Susan Bock, Matthew Weston, Keith P. George, Alan M. Batterham

**Affiliations:** 1 Health and Social Care Institute, Teesside University, Middlesbrough, United Kingdom; 2 School of Applied Social Sciences, Durham University, Durham, United Kingdom; 3 School of Social Sciences, Business & Law, Teesside University, Middlesbrough, United Kingdom; 4 Research Institute for Sport and Exercise Sciences, Liverpool John Moore’s University, Liverpool, United Kingdom; TNO, NETHERLANDS

## Abstract

**Background:**

Low-volume high-intensity interval training holds promise for cardiometabolic health promotion in adolescents, but sustainable interventions must be practical and engaging. We examined the effect of a school-based multi-activity low-volume high-intensity interval training intervention on adolescents’ cardiometabolic health.

**Methods:**

In an exploratory controlled before-and-after design, 101 adolescents (mean age ± standard deviation [SD] 14.0 ± 0.3 years) were recruited from four schools; two were designated as intervention sites (n = 41), and two as control (n = 60). The intervention comprised 4 to 7 repetitions of 45 s maximal effort exercise (basketball, boxing, dance and soccer drills) interspersed with 90-s rest, thrice weekly for 10 weeks. Outcomes were non-fasting blood lipids and glucose, waist circumference, high sensitivity C-reactive protein, resting blood pressure, physical activity, twenty-metre shuttle-run test performance and carotid artery intima-media thickness. The difference in the change from baseline (intervention minus control) was estimated for each outcome. Using magnitude-based inferences, we calculated the probability that the true population effect was beneficial, trivial, and harmful against a threshold for the minimum clinically important difference of 0.2 between-subject SDs.

**Results and Discussion:**

Mean (± SD) attendance for the intervention (expressed as percentage of available intervention sessions [n = 30]) was 77 ± 13%. Post-intervention, there were likely beneficial effects for triglycerides (-26%; 90% confidence interval -46% to 0%), waist circumference (-3.9 cm; -6.1 cm to -1.6 cm) and moderate-to-vigorous physical activity (+16 min; -5 to 38 min), and a possibly beneficial effect for twenty-metre shuttle-run test performance (+5 shuttles; -1 to 11 shuttles) in intervention participants (vs controls). The role of elevated triglycerides and waist circumference in cardiovascular disease and metabolic syndrome development underlines the importance of our findings. We also demonstrated that school-based low-volume high-intensity interval training can be delivered as intended, thus representing a novel and scalable means of improving aspects of adolescents’ cardiometabolic health.

**Trial Registration:**

ClinicalTrials.gov NCT02626767

## Introduction

The onset of cardiometabolic risk factor clustering begins early in life [1], and can manifest as the paediatric metabolic syndrome—a combination of risk factors for cardiovascular disease and type 2 diabetes. These factors include abdominal obesity, hypertension, glucose intolerance, elevated triglycerides and decreased high-density lipoprotein (HDL) cholesterol [2]. Whilst high levels of cardiorespiratory fitness and physical activity are likely cardioprotective [3], recent findings suggest these outcomes are in decline in English youth [4,5]. Further, despite an apparent plateau in obesity rates [6], English adolescents’ waist circumferences have substantially increased over the last 35 years [7]. These unfavourable changes provide clear justification for the development of interventions targeting modifiable cardiometabolic risk factors in youth.

Often, exercise interventions involving young people have focused on increasing moderate-to-vigorous physical activity (MVPA); however, low levels of MVPA in adolescents has led to suggestions that this population might have difficulty, and perhaps little interest, in engaging in activity of this kind [8]. As such, previous intervention efforts with adolescents may have failed due to a mismatch between the intervention activities and what participants actually want to do. There is also accumulating evidence from both cross-sectional [9] and longitudinal datasets [10] that it is vigorous- not moderate-intensity activity that is associated with lower measures of waist circumference, systolic blood pressure and body mass index (BMI) in youth. This association remains despite vigorous activity only occupying a small proportion of young peoples’ total physical activity per day (~4 minutes) [10]. With this is mind, high-intensity interval training (HIT)—characterised by short, intermittent bursts of vigorous activity, alternated with periods of rest or low intensity active recovery [11]—may represent a potential alternative to ‘traditional’ MVPA programmes. Low-volume HIT typically involves ~30 to 60 s activity bursts performed at either “all-out” (e.g. sprints) or maximal effort intensity (i.e. ≥90% of peak oxygen uptake [VO_2peak_]/90-95% of maximum heart rate [HR_max_]) [12], which necessitates work to rest ratios of ≤1, and a short total exercise duration [13]. Over the last decade, there has been renewed scientific interest in the efficacy of low-volume HIT as a time-efficient means of improving health and fitness markers, such that there is now strong evidence that it can enhance outcomes such as cardiorespiratory fitness [13,14] and insulin sensitivity [15,16,17]. Many of these findings are confined to adults, however, with the effects of low-volume HIT in young people still relatively under researched [18,19].

Recently, Costigan et al. [18] meta-analysed the effect of eight youth-based HIT studies utilising various protocols, populations and outcomes, and found large effects for cardiorespiratory fitness (unstandardized mean difference 2.6 ml.kg^-1^.min^-1^; 95% confidence interval 1.8 to 3.3 ml.kg^-1^.min^-1^; effect size 1.05). In a narrative review of 11 studies, Logan et al. [19] reported improvements in VO_2peak_, insulin sensitivity and HDL cholesterol, and reductions in percentage body fat, systolic blood pressure, waist circumference, fasting blood glucose, low-density lipoprotein (LDL) cholesterol and triglycerides in adolescents following various HIT protocols. Nonetheless, to fully elucidate the impact of low-volume HIT on health, fitness and physical activity outcomes in adolescents, more research is required [19].

In recent years, the concept of embedding HIT within the school day has begun to be explored. Several school-based HIT trials have utilised running- [8,20,21] or cycle ergometry-based [22] protocols. In light of recent calls for HIT models to include a variety of engaging activities, however [23], it is questionable whether programmes based exclusively on one ‘traditional’ exercise mode would hold sustained appeal for diverse adolescent groups, particularly adolescent girls [24]. Further, regardless of outcome, protocols requiring specialist equipment like cycle ergometers could simply be deemed impractical in real-life settings like schools, due to costs. This issue was partly addressed in a recent trial in New Zealand, where 8-weeks of twice-weekly HIT, performed on pre-existing school physical education (PE) equipment such as rowing machines, treadmills and cross trainers, yielded improvements in VO_2peak_, body fat percentage, lean tissue mass, visceral fat mass and waist circumference-to-height ratio in a small group of low-active male adolescents [25]. Here, however, HIT was supplemented with resistance training; thus determining the isolated effect of HIT was not possible. More recently, Costigan et al. [26] reported high levels of study acceptability and moderate intervention effects for waist circumference (-1.5 cm; 95% C.I -3.4 to 0.4 cm; compared to controls) in 21 adolescents from one Australian school, following an 8-week HIT programme based on activities such as shuttle runs, jumping jacks and skipping. Nonetheless, the authors conceded that larger numbers of participants from multiple schools were required to fully examine the feasibility of embedding HIT into school settings. We aimed to examine the effect of a school-based multi-activity low-volume HIT intervention (named Project FFAB [Fun Fast Activity Blasts]) on cardiometabolic risk factors in English adolescents.

## Methods

### Study design

Ethics approval for Project FFAB was obtained from the Teesside University Research Governance and Ethics Committee (reference number 008/11), and the study was conducted in accordance with the Declaration of Helsinki. In January 2011, eight secondary schools in the Tees Valley area of Northeast England were invited to take part in the study. The head teachers of four schools provided written informed consent. Using an exploratory controlled before-and-after study design (clustered), two schools were designated as intervention sites, and two as control. Schools were broadly equivalent for the relative deprivation of the neighbourhood in which they were situated, such that in each arm of the trial one school was in the top quintile and the other in the bottom quintile for the Index of Multiple Deprivation [27]. The study took place from March 2011 to June 2011, and the protocol registered retrospectively on clinicaltrials.gov (trial number NCT02626767) in December 2015. We did not register the trial prospectively, as at the time we did not view registration as a requirement for a non-randomised (i.e., observational) study. The design, conduct and reporting of the trial adheres to the Transparent Reporting of Evaluations with Non-randomised Designs statement [28] (S1 Text) and we confirm that the trial is reported per the original protocol (S2 Text), with no selective reporting of outcomes or outcome switching, bar the omission of LDL cholesterol measures for reasons described subsequently.

**Fig 1:**
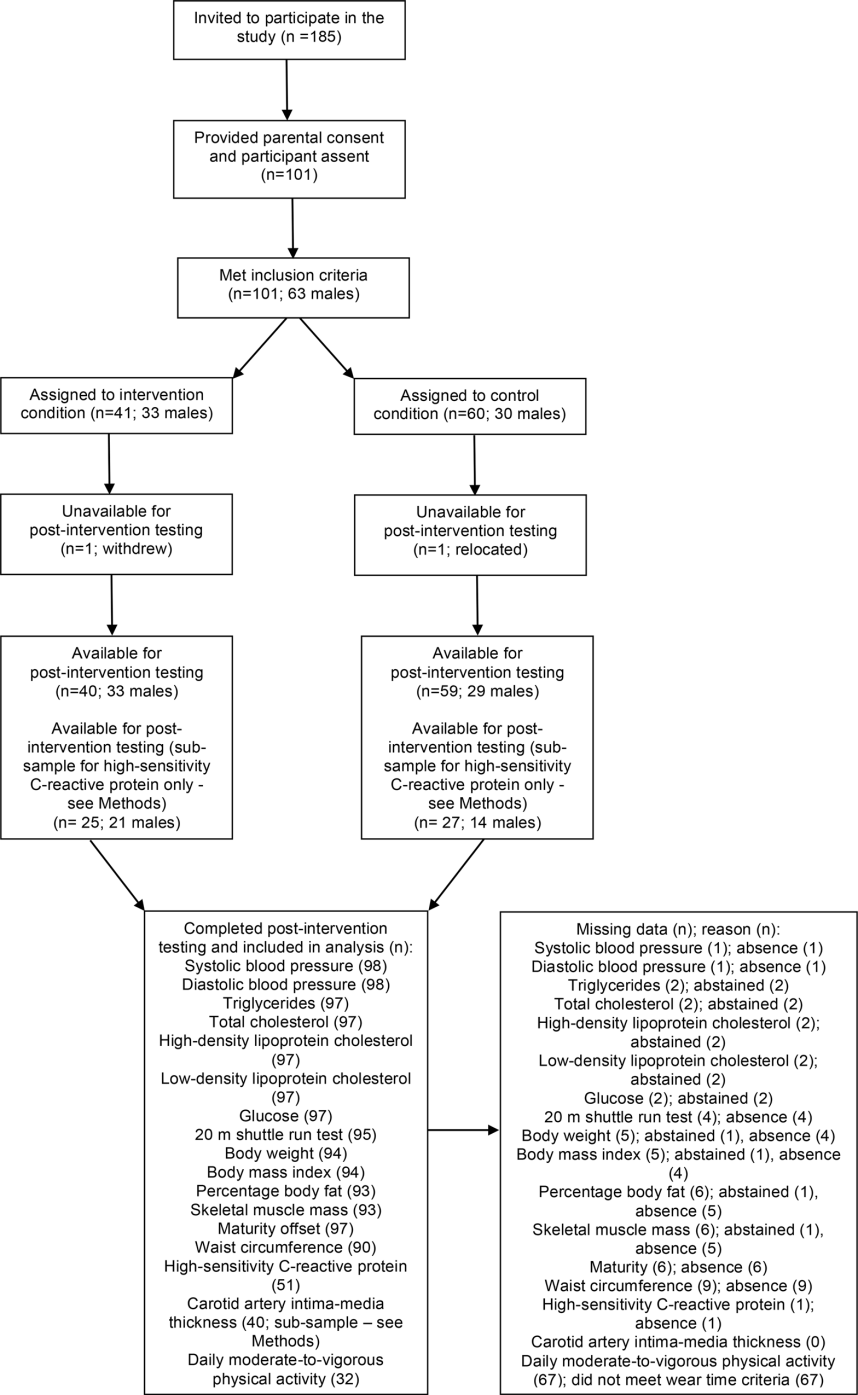
Participant flow-chart.

Trial recruitment took place in February 2011 during PE lessons for Year 9 school pupils (aged 13 to 14 years). The first author (KLW) delivered a short presentation about the study, and then distributed packs containing information sheets, physical activity readiness questionnaires, parental consent and participant assent forms to all pupils in attendance (n = 185 across four schools). In line with the Medical Research Council’s guidance on developing and evaluating complex interventions [29], Project FFAB was defined as an exploratory trial. Our target sample size was 100 Year 9 pupils (~25 participants per school), which would inform a future definitive trial by examining whether the intervention could be delivered as intended, with regards to compliance and retention [29]. Pupils were eligible to participate if were in Year 9, free from exclusion criteria and had provided written informed parental consent and participant assent. Exclusion criteria were symptoms of or known presence of heart disease or major atherosclerotic cardiovascular disease, condition or injury or co-morbidity affecting the ability to undertake exercise, diabetes mellitus, early family history of sudden cardiac death, condition or disorder which is communicable via blood, and pregnancy or likelihood of pregnancy. Of the 185 pupils receiving study information packs, 101 (62 males; aged 14.1 ± 0.3 years [mean ± SD]) provided written informed parental consent and participant assent (55% recruitment rate), of which 41 (33 males) attended intervention schools (Fig 1).

### Intervention protocol

The Project FFAB intervention is described according to the requirements of the Template for Intervention Description and Replication (TIDiER) checklist [30] (S3 Text). The intervention took place over 13 calendar weeks, which incorporated the 10-week intervention, a 2-week Easter holiday (occurring after intervention week 4), and a 1-week mid-term holiday after week 9. The timing and length of school holidays were uniform across the four schools. At intervention schools, the intervention replaced normal PE lessons. Control participants continued with their usual PE curriculum throughout the intervention period and were not made aware of what Project FFAB entailed at other schools.

The intervention took place thrice weekly and was delivered by the first author (KLW) who has extensive experience in exercise delivery and instruction. Participants performed two low-volume HIT sessions per week during PE lessons, with the third completed after school or during the school lunch break in the sports hall. At both schools, participants chose the timing of the third session. Participants attending intervention school number one attended sessions as one group of 24 males. At intervention school two, participants completed mixed-sex sessions in one group of 8 (5 females) and one group of 9 (3 females), owing to their scheduled PE lessons taking place on different days. Participants were encouraged to attend as many sessions as possible; those who completed ≥70% were awarded a t-shirt, with individuals attending ≥90% also entered into a prize draw to win a pair of training shoes.

The low-volume HIT sessions commenced with a 5-min warm-up and culminated with a 5-min cool down. Following the warm-up, participants performed four repetitions of 45-s of maximal effort exercise (basketball, boxing, dance and soccer drills; examples of which can be found in Table 1), each interspersed with 90-s recovery. Activities were chosen based on qualitative data collected in pre-intervention focus groups with adolescent school students. This approach, often referred to as formative research, has been shown to effectively inform the development of physical activity interventions [24]. In our study, the focus groups were conducted to aid the development of the intervention and maximise the likelihood of participants’ attendance and compliance with the low-volume HIT sessions. During the focus groups, participants expressed a desire for the intervention to incorporate a variety of activities (namely, boxing, dance and soccer), with the exercise mode rotated frequently. Data collected during a pilot of Project FFAB confirmed that 45-s drills based on boxing, dance and soccer were capable of eliciting a high-intensity dose (e.g. peak heart rate ≥90% of maximal) [31]. As such, the activities constituting the low-volume HIT sessions changed on a weekly basis, with the number of repetitions performed during each session increasing from four to seven across the 10-week intervention. Equipment required for the sessions (e.g. soccer balls, basketballs and music system) were already available at the intervention schools. The exception to this was the boxing equipment, which was provided by Teesside University.

**Table 1:** Example drills from the low-volume high-intensity interval training sessions.

**Activity**	**Drills**
Boxing	Fast jabs on the focus pads
Boxing	Ten jabs on the focus pads, then perform five star jumps
Boxing	Ten jabs on the focus pads, then run to the end of the sports hall and back
Boxing	Ten fast side steps dodging the focus pads, then run to the end of the sports hall and back
Boxing	Five combination punches (hook and jab) on the focus pads, then run to the end of the sports hall and back
Basketball	Receive and return a chest pass, then run to a cone and back
Basketball	Run round a square course, and receive and return a bounce pass on one corner of the square
Basketball	Bounce a basketball five times, then run to the end of the sports hall and back
Basketball	Receive and return a shoulder pass, then run to a cone and back
Dance	Jump up and down whilst waving pom poms above head height
Dance	Perform star jumps whilst waving pom poms.
Dance	Stationary high-knees runs
Dance	Fast side kicks
Dance	High leg kicks whilst clapping pom poms underneath elevated leg
Soccer	Kick a soccer ball into a goal, then run to end of the sports hall and back
Soccer	Perform ten toe touches on a soccer ball, then run to a cone and back
Soccer	Perform fast feet movements through cones setup, then run to end of the sport hall and back
Soccer	Jump up to head a soccer ball five times, then run to the end of the sports hall and back
Soccer	Running round the sports hall following a square or diagonal course

At the start of the PE-based sessions, participants were fitted with a heart rate monitor (Polar RS400, Polar Electro, Finland). Due to the larger group size at intervention school one (n = 24), participants only wore monitors for one PE-based session per week, whereas participants at intervention school two (n = 17; split across two groups), wore monitors during every PE-based session. To minimise participant burden, heart rate data were not collected at non-PE sessions. A full description of the heart rate data collection, reduction and analysis has been reported previously [32]. To summarise, a cut-point of ≥90% of maximal heart rate was used as our criterion for satisfactory compliance to high-intensity exercise, reflecting that used in previous work [12]. Accordingly, participants were verbally motivated to provide “maximal efforts” and reach ≥90% of their maximal heart rate on each 45-s repetition. To encourage intensity compliance, we checked participants’ heart rates during each low-volume HIT session. Afterwards, we derived the peak heart rate of each 45-s exercise repetition from each individual file using the Polar ProTrainer software (Polar Electro, Kempele, Finland), which were expressed and recorded as a percentage of the participant’s maximal heart rate. Participants’ maximal heart rates were determined as the highest 5-s value recorded during the low-volume HIT sessions, or the baseline twenty-metre shuttle-run test.

### Outcome measures

Outcome measures were collected during lesson time allocated for PE, by trained research assistants at baseline (February 2011), and up to seven days post-intervention (June 2011). All blood samples taken post-intervention were collected four days after the last exercise session. Due to the nature of the intervention, it was not possible to blind participants to group condition. The timing of data collection across the four schools was uniform at baseline and post-intervention. Due to resource limitations, two outcomes (high sensitivity C-reactive protein [hsCRP] and carotid artery intima-media thickness [cIMT]) were measured in a subset of participants (n = 53 [hsCRP] and n = 40 [cIMT]) only. All participants were instructed not to modify their dietary or lifestyle habits during the trial period and received a “thank you” pack for their involvement at the end of the study.

#### Anthropometric and maturity assessments

Using calibrated scales (Seca, Birmingham, UK) and a portable stadiometer (Leicester Height Measure, Seca, Birmingham, UK), body mass, stature and sitting height were measured to the nearest 0.1 kg and 0.1 cm, respectively. During assessments, participants wore light PE clothing and were barefoot. Two measurements were taken for stature and sitting height, with a third obtained if the first two measurements differed by ≥0.4 cm. Measurements were then averaged; in the case of three measurements the median value was used. Leg length was calculated by subtracting sitting height from stature. Somatic maturity was estimated for each participant by predicting years from attainment of peak height velocity via sex-specific multivariable equations that included stature, sitting height, leg length, body mass, chronological age and their interactions [33]. Body mass index was calculated as weight in kilograms divided by height in metres squared, and participants classified as underweight, normal weight or overweight using international sex-specific cut-points [34]. Skeletal muscle mass and percentage body fat were estimated using the InBody 720 (Biospace, Gateshead, UK); an octopolar tactile-electrode bioelectrical impedance analyser (Biospace, Gateshead, UK) used to measure body composition in youths due to its high precision [35]. Waist circumference was measured using a non-elastic Gulick tape measure (G-tape) with a compression spring tension device on the participants’ bare midriff midway between the tenth rib and the iliac crest. The measurement was taken at the end of a gentle expiration and recorded to the nearest 0.1 cm on three occasions. The average of the first two measures within 1 cm was used for analysis.

#### Blood profiling and blood pressure

Blood lipids, glucose and hsCRP profiles were assessed via the Cholestech LDX analyser (Cholestech Corporation, Hayward, CA, USA), which is a reliable and valid alternative to gold standard methods for cardiovascular risk screening [36,37]. Due to accumulating evidence that fasting prior to sampling does not result in clinically significant differences in lipid levels compared to non-fasted samples [38,39], and suggestions that non-fasting lipid values might be more representative of usual metabolic conditions [39], participants were not asked to fast before testing. Instead, we recorded the number of hours since participants had last consumed any food or drink other than water, and included this variable as a covariate in our statistical analysis. Before each measurement session, an optics check was performed on the LDX. Capillary blood was collected using a finger prick method to obtain values of plasmatic total cholesterol, HDL cholesterol, triglycerides and glucose. We elected not to include LDL cholesterol, as the Friedewald equation used to estimate it assumes a constant triglyceride: cholesterol ratio in very low-density lipoprotein particles that does not hold in the non-fasting state [40]. Samples were drawn into a 35 μL capillary tube (Cholestech LDX, AR-MED Ltd, Egham, UK), immediately transferred into the sample well of a lipid profile and glucose cassette (Cholestech LDX, AR-MED Ltd, Egham, UK) then placed in the analyser drawer. To obtain hsCRP profiles, the sampling process was repeated using a further 50 μL of blood and an hsCRP cassette (Cholestech LDX, AR-MED Ltd, Egham, UK). All cassettes were stored in a refrigerator and brought to room temperature at least 15 minutes before use. Systolic and diastolic blood pressures were measured using the Omron MX3 Plus monitor (Model HEM-742-E; Omron Healthcare UK, Milton Keynes, UK). Seated measurements were taken after participants had rested for at least five minutes. A minimum of two readings were obtained and averaged for analysis.

#### Carotid artery intima-media thickness

One trained ultrasound technician performed all cIMT measurements. Two-dimensional (B-mode) imaging scans were performed using a standard ultrasound system (Mylab30CV system, ESAOTE, Italy) with a 10 MHz linear phased array transducer. All participants were assessed in the seated position. Ten millimetre segments of the far wall of the right common carotid artery, 1 to 2 cm proximal to the carotid bulb were imaged. Care was taken to generate clear images of the carotid intima media by optimal adjustment of depth, gain and filters. Four images were digitally recorded and analysed off-line (IMT.LAB version 1.1, Pie Medical Equipment, Netherlands) by a single technician blinded to the group condition. The software allowed manual checking of the distance between interfaces of the lumen-intima and media-adventitia and generated data for mean cIMT.

#### Twenty Metre shuttle-run test performance

Twenty-metre shuttle-run test (20mSRT) performance was assessed indoors on hard-floored sports halls, using the British National Coaching Foundation protocol [41]. Participants’ heart rates were recorded via Polar RS400 monitors (Polar, Kempele, Finland) at 5-s intervals throughout the 20mSRT. Afterwards, the data were downloaded into the Polar ProTrainer 5 software (Polar, Kempele, Finland) and the peak 5 s heart rate attained during the 20mSRT for each participant was recorded. The mean (±SD) peak heart rate for intervention and control participants was 203 ± 8 beats·min^-1^ and 206 ± 4 beats·mins^-^1, respectively. Test performance was expressed as the number of shuttles completed.

#### Physical activity

Physical activity was measured via Actigraph GT1M accelerometers (Actigraph, LLC, Pensacola, Florida), which were initialised to collect data at 10-s epochs using ActiLife software (Version 5.8.3). Participants were instructed to wear their device on their right hip during all waking hours, except when engaging in water-based activities, for seven consecutive days prior to the intervention, and for seven days after the intervention finished. Data were analysed using ActiLife software (Version 5.8.3), with non-wear time calculated as periods of 20 minutes or more of consecutive zero accelerometer counts [42]. Further analyses were only completed for participants who had worn their accelerometer for at least four days, with a minimum of 10 hours (600 minutes) recorded per day [43]. To estimate time spent performing different intensities of activity (presented as the average number of minutes per day), we applied the cut-points developed by Evenson et al. [44], based on recommendations by Trost et al. [45].

### Statistical Analysis

All blood measures were log-transformed prior to analysis. Accordingly, the descriptive summary for these variables comprises the geometric mean, with the dispersion shown as a ×/ ÷ factor standard deviation (SD) [46]. For all other outcomes, the descriptive summary comprises arithmetic means ± SD. Residual plots (not shown) were visually inspected for all analyses, to check that the models were correctly specified (uniform variance and normal distribution of residuals).

Outcome data were analysed using an analysis of covariance (ANCOVA) model. The independent variable was the group (intervention or control), with the dependent variable as the post-intervention value. Model covariates were sex, maturity offset, and baseline value of the outcome, to control for any imbalances between the intervention and control groups at baseline [47]. For the blood lipid and glucose measures only, fasting status was included as an additional covariate. This variable was defined as the number of hours fasted post-intervention minus number of hours fasted at baseline. Strictly, shuttle run performance is a count outcome, and should be modelled using a Poisson or negative binomial distribution. However, residuals plots revealed that treating number of shuttles as a normal variable was robust, and this better facilitated subsequent inference. Using a magnitude-based inferences framework [46,48], the mean effect of the intervention (versus control) for each outcome was presented together with the uncertainty of the estimates expressed as 90% confidence intervals. Log-transformed variables were back transformed to obtain the percent difference between groups. The adjusted mean intervention effects were evaluated for their practical/ clinical significance by pre-specifying the minimum clinically important difference (MCID) [49]. In the absence of a robust clinical anchor, the MCID is conventionally defined using a distribution-based method as a standardised mean difference of 0.2 between-subject standard deviations (SD) [50]. The SD of the pooled baseline values was used for this purpose, as the post-intervention SD can be inflated by individual differences in responses to the exercise intervention. Using the mean intervention effect for each outcome, together with its uncertainty, the probability (percent chances) that the true population effect was beneficial (>MCID), harmful (>MCID with opposite sign), or trivial (within ± MCID) was calculated [46]. Using clinical inferences, qualitative probabilistic terms were assigned to each effect using the following scale; <0.5%, most unlikely or almost certainly not; 0.5 to 5%, very unlikely; 5 to 25%, unlikely or probably not; 25 to 75%, possibly; 75 to 95%, likely or probably; 95 to 99.5%, very likely; >99.5%, most likely or almost certainly [46]. In line with the recommendations by Hopkins et al. [46], a clinically unclear effect is possibly beneficial (>25%) with an unacceptable risk of harm (>0.5%) and an odds ratio for benefit: harm of <66; all other effects are clear.

In this exploratory study, there are too few clusters (schools) per group to permit robust modelling of the hierarchical data structure or indeed estimates of the clustered standard error for the mean intervention effect. Therefore, data were analysed at the individual level, with 90% confidence intervals for the intervention effect derived by multiplying the obtained standard error by the appropriate value of the t-distribution with just 2 degrees of freedom (given two clusters in each arm of the study).

With the current design, cases with missing post-intervention data contribute no information regarding the intervention effect; therefore these cases were removed from the analysis. However, there were several participants with observed values of the post-intervention outcome but missing baseline value and/ or other covariate data. For example, post-intervention waist circumference data were available for 90/101 participants, but either baseline waist circumference and/ or maturity offset data were missing in 6 cases (84 complete cases). Participants with observed post-intervention data but missing covariates do indeed contribute information about the intervention effect and should be included in the analysis according to the intention-to-treat principle. Assuming the missing baseline data were missing at random, we included these incomplete cases in the ANCOVA analysis model by applying a principled method–full-information (direct) maximum likelihood [51]—using the Stata® SEM module (v. 13.1; Stata Corp. College Station, Texas, USA). This method derives the parameter estimates that, if true, would maximise the probability of having observed the data at hand.

In addition to the missing data, we observed values below the lower detection limit of the Cholestech LDX analyser for a substantial number of the blood measures for before and/ or after the intervention: n = 56 for hsCRP (<0.31 mg/L), 37 for triglycerides (<0.51 mmol/L), 3 for HDL cholesterol (<0.39 mmol/L), and 2 for total cholesterol (<2.59 mmol/L). These left-censored values are not missing data, and we applied a principled method–multiple imputation—to include them appropriately in the analysis [52]. Using interval regression with chained equations in Stata® software, we imputed left-censored values for both pre- and post-intervention measurements between the lower detection limit and fractionally above zero, conditional on sex, maturity offset, plus the fasting status covariate for all variables except hsCRP. One hundred imputations were made. The ANCOVA analysis detailed above was then applied to the 100 imputed data sets with the results combined using Rubin’s rules [53].

Peak heart rate data (percentage of maximal) from the attended low-volume HIT sessions were analysed via proportion analysis and linear mixed modelling, the process of which has been published elsewhere [32]. Briefly, we determined the proportion of repetitions in which the high-intensity exercise criterion was attained for each participant; then derived the median and interquartile range of these individual proportions. We then applied a linear mixed model with sex, session, and repetitions (nested within a session) included as fixed effects to provide the correct overall between- and within-subject variability (expressed as an SD) in peak heart rate across the repeat 45-s repetitions. These data are expressed as mean ± SD, with uncertainty in the estimates expressed as 95% confidence intervals.

## Results

### Low-volume HIT sessions

During the Project FFAB intervention, 159 high-intensity exercise repetitions were delivered across 30 sessions at each intervention school. The total exercise time commitment was 419 minutes and 15 seconds, inclusive of warm-up and cool-down activities. The amount of high-intensity work was therefore 119 minutes 15 seconds (~12 minutes per week). One female participant dropped out after week 6, citing a lack of interest. Two male participants sustained injuries unrelated to the study after weeks 5 and 7, therefore did not complete the remaining low-volume HIT sessions. Of the 38 participants that completed the intervention, mean (±SD) attendance (expressed as a percentage of total sessions) was 77±13%. Reasons for participant absence were illness, individual family holidays; and for the non-PE based sessions, prior commitments or forgetfulness. Of the attended sessions, the median (interquartile range) of the proportions of repetitions for individual participants wherein the high-intensity exercise criterion was attained was 69% (43% to 80%). The mean for peak heart rate across all repetitions was 91% of maximal. The mixed model analysis revealed that the between- and within-subject SDs were 3.4 (95% confidence interval 2.7 to 4.3) percentage points and 4.3 (4.1 to 4.4) percentage points, respectively.

## Descriptive Summary Data

Descriptive data of the participants’ baseline characteristics are shown in Table 2 (S1 Table). Using international youth cut-points for BMI [31], 8% of participants were underweight, 70% normal weight and 22% overweight or obese; and 35% of participants (19% of intervention participants) had a waist circumference greater than the 90^th^ percentile for 14 year old British adolescents (70.6 cm for girls and 76.1 cm for boys; [54]). Two males declined blood profiling post-intervention, and one female abstained from body composition assessment throughout. Other missing data were due to participants’ absence from school on days which data collection took place. One control participant relocated during the study and one intervention participant withdrew due to lack of interest; therefore post-intervention data were unavailable for these participants. Both participants who sustained injuries during the study period completed post-intervention data collection. The participant flow and numbers included in the analysis for each outcome are summarised in Fig 1.

**Table 2:** Participants' baseline characteristics. BMI = body mass index. SMM = skeletal muscle mass. WC = waist circumference. BP (sys or dia) = blood pressure (systolic or diastolic). 20mSRT performance = 20m shuttle-run test performance. cIMT = carotid artery intima-media thickness. Daily MVPA = daily moderate-to-vigorous physical activity. TG = triglycerides. TC = total cholesterol. HDL = HDL cholesterol. GLU = glucose. hsCRP = high-sensitivity C-reactive protein.

**Variable**	**Control (n = 60)**	**Intervention (n = 41)**
	Arithmetic mean ± SD	
Sex (male/female) n	30/30	33/8
Age (years)	14.1 ± 0.3	14.1 ± 0.3
Maturity offset (years)	0.5 ± 1.3	0.3 ± 1.0
Height (cm)	163.6 ± 6.9	165.7 ± 7.2
Weight (kg)	55.3 ± 9.2	60.2 ± 15.3
BMI (kg/m^2^)	20.5 ± 2.7	21.8 ± 4.5
Body fat (%)	19.6 ± 7.8	18.3 ± 11.1
SMM (kg)	24.3 ± 4.4	26.7 ± 5.2
WC (cm)	70.0 ± 8.8	77.4 ± 13.7
BP (sys) (mmHg)	118 ± 10	122 ± 11
BP (dia) (mmHg)	68 ± 8	72 ± 9
20mSRT performance (Number of shuttles)	60 ± 23	49 ± 22
cIMT (mm)	0.40 ± 0.05*	0.40 ± 0.05*
Daily MVPA (mins)	73 ± 33	58 ± 18
	Geometric mean, ×/ ÷ SD	
TG (mmol/L)	0.79 ×/÷ 1.83	0.77 ×/÷ 1.91
TC (mmol/L)	3.67 ×/÷ 1.19	3.81 ×/÷ 1.18
HDL (mmol/L)	1.36 ×/÷ 1.39	1.34 ×/÷ 1.48
GLU (mmol/L)	5.29 ×/÷ 1.16	5.39 ×/÷ 1.13
hsCRP (mg/L)	0.35 ×/÷ 2.11**	0.33 ×/÷2.11**

* = based on subsample of participants n = 40 (23 intervention)

** = based on subsample of participants n = 53 (26 intervention)

### Post-intervention effects

ANCOVA adjusted post-intervention effects for each outcome are shown in Table 3 (S1 Dataset). When the intervention group was compared to controls, there was a likely beneficial effect for triglycerides, waist circumference and MVPA, and a possibly beneficial effect for 20mSRT performance. There were no clinically substantial effects for any other outcome.

**Table 3:** Mean post-intervention values adjusted for sex, baseline value and maturity offset. CON = control group. INT = intervention group. TG = triglycerides. WC = waist circumference. Daily MVPA = daily moderate-to-vigorous physical activity. 20mSRT = 20m shuttle-run test performance. BP (sys or dia) = blood pressure (systolic or diastolic). TC = total cholesterol. HDL = HDL cholesterol. cIMT = carotid artery intima-media thickness. Body fat (%) = percentage body fat. SMM = skeletal muscle mass. hsCRP = high-sensitivity C-reactive protein. BMI = body mass index. GLU = glucose.

**Variable**	**CON**	**INT**	**Difference**	**90% Confidence interval**	**Clinical Inference**
TG (mmol/L)*$	0.97	0.72	-26%	-46% to 0%	Likely beneficial, very unlikely harmful
WC (cm)	78.1	74.2	-3.9	-6.1 to -1.6	Likely beneficial, very unlikely harmful
Daily MVPA (min)	57	73	+16	-5 to 38	Likely beneficial, very unlikely harmful
20mSRT (Shuttles)	55	60	+5	-1 to 11	Possibly beneficial, very unlikely harmful
BP (sys) (mmHg)	119	117	-2	-9 to 5	Unclear
TC (mmol/L)*$	3.67	3.57	-3%	-9% to 4%	Unclear
HDL (mmol/L)*$	1.12	1.20	+7%	-14% to 34%	Unclear
hsCRP (mg/L)*	0.223	0.239	+7%	-40% to 100%	Unclear
cIMT (mm)	0.415	0.408	-0.007	-0.059 to 0.046	Unclear
Body fat (%)	20.2	19.6	-0.6	-2.7 to 1.5	Very unlikely harmful, unlikely beneficial
SMM (kg)	25.0	25.3	+0.3	-0.4 to 1.1	Very unlikely harmful, unlikely beneficial
Weight (kg)	58.2	58.5	+0.3	-1.4 to 1.9	Very unlikely harmful, very unlikely beneficial
BMI (kg/m^2^)	21.3	21.2	-0.16	-0.73 to 0.41	Very unlikely harmful, very unlikely beneficial
BP (dia) (mmHg)	64	64	+0.3	-3.9 to 4.6	Unlikely harmful, unlikely beneficial
GLU (mmol/L)*$	5.26	5.29	+0.7%	-7.5% to 9.6%	Unlikely harmful, unlikely beneficial

* = Back transformed geometric means derived from a log transformed analysis

$ = Adjusted for fasting status

## Discussion

Efforts to embed low-volume HIT into the school setting have delivered some promising findings to date; however recruitment of small and/or specific population samples (e.g. low-active males [25]) and adoption of single-activity protocols [8,20,21,22] hinders generalisability and application in wider youth populations and contexts. Further, when compared to the wealth of adult data on the topic, evidence demonstrating the effectiveness of low-volume HIT on health and fitness outcomes in adolescents is still lacking. The aim of our study therefore, was to examine the effects of Project FFAB—a 10-week school-based multi-activity low-volume HIT intervention—on cardiometabolic risk factors in English adolescents. Following Project FFAB, likely beneficial and possibly beneficial effects were observed in intervention participants (compared to controls) for triglycerides, waist circumference and daily MVPA, and 20mSRT performance, respectively. Our findings thus provide further evidence supporting the use of low-volume HIT to improve aspects of cardiometabolic health in adolescents.

It has previously been documented that low-volume HIT can decrease adolescents’ triglyceride levels by around 5% [55]; however in this case the study sample was made up exclusively of obese girls (n = 11). Our findings therefore, go beyond earlier work by reporting substantial improvements in triglycerides (-26%) from a representative adolescent sample that consisted of ‘under’, ‘normal’ and ‘overweight/obese’ males and females. While the mechanisms behind HIT-induced decreases in triglycerides are unclear, recent work suggests this may be due to decreases in the postprandial lipaemic response [56,57] and/or increases in postprandial resting fat oxidation [58,59]. The former theory is in light of research by Thackray et al. [56], where changes in adolescent boys’ fasting plasma triglyceride concentration were small to moderate (mean difference -0.05 mmol/L; 95% confidence interval -0.11 to 0.01 mmol/L; effect size 0.40) following a single running-based HIT session (10 x 60 s efforts at maximal aerobic speed, each interspersed with 60-s recovery). More recently, Bond et al. [59] reported that a single HIT bout performed via cycle ergometry (8 x 60 s efforts at 90% of peak power, interspersed with 75 s recovery) increased resting postprandial fat oxidation in adolescent males and females (aged 13 to 14 years), in the four hours subsequent to consuming a high fat meal. As such, whilst it was not our intention to explore the mechanisms underpinning the triglyceride reductions in our study, it could be hypothesised that the chronic reductions occurred as a result of the acute responses detailed above. With regards to waist circumference, substantial post-intervention reductions of 3.9 cm were observed in intervention participants compared to controls, despite a lack of meaningful changes in BMI, percentage body fat and skeletal muscle mass. This effect is similar to earlier HIT work in obese adolescent females [55]. Whilst we do not currently have data to indicate a mechanism for the reduction in waist circumference we observed, it may be due to preferential effects of HIT on abdominal/ visceral adiposity. To explore this hypothesis and confirm our preliminary findings, future studies should look to include more precise measures of visceral fat.

In the meta-analysis by Costigan et al. [18] large effects for cardiorespiratory fitness following HIT were reported, whereas the Project FFAB intervention had a possibly small beneficial effect on 20mSRT performance. Our finding might be partly explained by the fact that all of the meta-analysed studies (n = 8) in [18] utilised a treadmill-based ramp protocol to assess cardiorespiratory fitness, rather than a field-based measure. Nonetheless, improvements in 20mSRT performance were observed following a 7-week low-volume HIT programme in Scottish adolescents [8,21]. Here, however, both the fitness test and HIT sessions were running-based, whereas in Project FFAB the exercise mode of the fitness test and intervention activities differed. As performance improvements are more likely in trials where strong similarities (specificity) between the exercise testing and training sessions exist [60], this might further explain our findings. With regards to daily MVPA, we observed a likely beneficial 16-minute increase in intervention participants compared to controls. This finding was not entirely unsurprising, as it has recently been speculated that school-based HIT may actually encourage participants to self-select higher levels of physical activity outside of the school environment [20]. Nevertheless, in the trial from which this claim was made, physical activity data were collected via self-report [20]. Project FFAB, therefore, is the first study to demonstrate that forms of low-volume HIT can substantially improve adolescents’ daily levels of MVPA when assessed using objective measures (accelerometry). Whilst this finding is no doubt promising, we did experience instances where participants failed to meet our accelerometer wear time criteria (≥4 days wear time and ≥600 minutes recorded each day). At baseline, 61 participants met our wear time criteria, however this decreased to only 32 post-intervention. Although these compliance figures are not dissimilar to those reported elsewhere [43], it is possible that this issue may have influenced our findings. As such, it is recommended that future studies aiming to build upon our preliminary observations for daily MVPA focus on engaging young people not only with the exercise intervention, but also the methods required to objectively assess physical activity.

### Limitations

Although our study has produced a number of promising findings, it is important to acknowledge a number of limitations. With non-randomised allocation, selection bias (where systematic differences in the treatment groups arise at baseline) might have occurred. Nonetheless, this threat to validity was at least partly assuaged by the equivalence of the schools between study arms for relative level of deprivation, and by adjusting for baseline imbalance in the statistical analysis. We also attempted to account for the clustering of pupils within schools by using a relatively conservative estimation of confidence intervals for magnitude-based inferences. However, with only two schools each in the intervention and control caution is warranted in study inferences, as appropriate for an exploratory controlled before-and-after study. In addition, as only eight females took part in the Project FFAB intervention, the feasibility of multi-activity low-volume HIT in adolescent females remains relatively unclear at this time. It is important to note, however, that the lack of girls in our intervention arm was not solely due to disinterest from potential female participants during the recruitment phase. Rather, the sex imbalance can be attributed to the fact that one intervention school only permitted access to one PE class for recruitment, of which all pupils were males. As such, future HIT studies aimed at improving adolescent health should aim to recruit both males and females, especially when utilising multi-activity programmes designed to hold appeal for both sexes. Further, whilst we acknowledge that a large, definitive randomised trial conducted across multiple study centres is needed to confirm and extend our findings, we have shown that multi-activity low-volume HIT can successfully be delivered as intended with regards to implementation, attendance and retention within the school environment. This finding is also important, given that the feasibility of conducting HIT in non-laboratory, real-life settings has been strongly contested of late [61]. Finally, due to equipment availability it was not possible to monitor exercise heart rates during every low-volume HIT session across the intervention, and as such a full fidelity analysis across both schools was not possible. We have, nonetheless, provided a robust fidelity evaluation utilising data from one of our intervention schools elsewhere [32]. To overcome this limitation, future trials should consider the use of monitoring tools such as ratings of perceived exertion (RPE), which provide a simple, practical, inexpensive and valid method for measuring exercise intensity [62].

## Conclusions

The results of our study demonstrate meaningful improvements in triglycerides, waist circumference and daily MVPA levels of English adolescents following a novel school-based multi-activity low-volume HIT intervention. The role of elevated triglycerides and waist circumferences in the development of cardiovascular disease and the metabolic syndrome [2] underlines the relevance of our findings from a cardiometabolic health perspective. Further, our study represents the first low-volume HIT trial to report substantial post-intervention improvements in daily MVPA levels, using objective accelerometry. Project FFAB also goes beyond previous school-based HIT trials, by demonstrating the effectiveness of a low-volume HIT trial delivered across two different school sites, in which adolescents’ insights on intervention development were utilised to develop a novel multi-activity exercise programme that was well accepted and adhered to. As such, this type of intervention could represent a novel and scalable means of improving important aspects of adolescents’ cardiometabolic health within the school environment.

## Supporting Information

S1 Text Populated TREND statement checklist. (PDF)

S2 Text Study protocol. (DOC)

S3 Text Populated TIDiER checklist. (DOCX)

S1 Dataset Stata file.dta. (DTA)

S1 Table Baseline comparisons of retained participants (complete cases), and those lost to follow-up (incomplete cases). (DOCX)
