# Supplementary material for: Effect of Novel, School-Based High-Intensity Interval Training (HIT) on Cardiometabolic Health in Adolescents: Project FFAB (Fun Fast Activity Blasts) - An Exploratory Controlled Before-And-After Trial
Source: PLoS One. 2016 Aug 3;11(8):e0159116. doi: 10.1371/journal.pone.0159116 (PMC4972319; doi:10.1371/journal.pone.0159116)
Supplement: S2 Text — (DOC) [file pone.0159116.s002.doc]

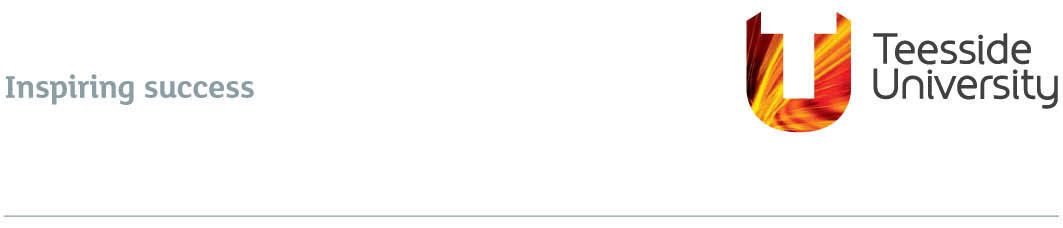


**Assessment of various health outcome measures and physical activity levels in Year 9 pupils.**

Investigators:

Kathryn Weston^1^

Prof Alan Batterham^1^

Dr Liane Azevedo^1^

Dr Susan Bock^2^

Dr Matthew Weston^3^

Prof Keith George^4^

^1^School of Health and Social Care, Teesside University

^2^Durham University

^3^School of Social Sciences, Business and Law, Teesside University

^4^Liverpool John Moores University

Contact:

Kathryn Weston

School of Health and Social Care

Teesside University

Middlesbrough

TS1 3BA

Phone: +44 (0) 1642 342988

Email: k.weston@tees.ac.uk

**Table of contents**

Appendix A: Protocol of the study

Appendix B: Head Teacher consent form

Appendix C: Parental consent form (control schools)

Appendix D: Parental consent form (intervention schools)

Appendix E: Young person assent form

Appendix F: Letter to head teacher

Appendix G: Head teacher information sheet (control schools)

Appendix H: Letter to the parents

Appendix I: Parent information sheet (control schools)

Appendix J: Child information sheet (control schools)

Appendix K: Head teacher information sheet (intervention schools)

Appendix L: Parent information sheet (intervention schools)

Appendix M: Child information sheet (intervention schools only)

Appendix N: Exercise and Physical Activity Readiness Assessment for Children and Adolescents

Appendix A: Protocol of the study

**Introduction**

Lack of physical activity has been linked with the metabolic syndrome which is being seen increasingly in children and adolescents (Weiss et al., 2004). Results from previous school-based physical activity interventions have been disappointing and a possible solution could be implementing an intervention where participants complete only a brief activity bout. Support for low volume high-intensity interval training (HIT) is growing; studies have shown that HIT can improve various metabolic and cardiovascular disease risk markers (Babraj et al., 2009; Tjønna et al., 2009; Whyte et al., 2010). Additionally, it has been suggested that brief maximal intensity exercise better resembles the activity patterns of adolescents, compared with longer, less intense bouts (Chia & Armstrong, 2007). Although this novel idea has not yet been fully explored in young people of both sexes, or in a school setting on a large scale; the growing body of work in HIT programmes is promising, and warrants future work to establish how these interventions may work in a ‘real life’ setting. The aim of this study therefore is to investigate whether a 10 week fun short physical activity blasts intervention substantially improves cardiometabolic risk factors and physical activity levels in Year 9 pupils. The study aim will be blinded to the school teachers, parents and young people to reduce substantial bias due to reactive behavioural effects; if the young people and parents and teachers know what the research question, they may alter behaviours during the testing period. However, all the procedures will be fully explained and the findings will be briefed after the study.

**Methods**

***Design***

The study is an exploratory controlled before-and-after trial with two parallel groups. Participants are 13-14 year old school pupils drawn from Year 9 Physical Education classes at four local schools. The trial will utilise a cluster design to avoid contamination. Thus, schools will be allocated to one of two groups. Two schools will be the intervention group where participants will take part in fun short physical activity blast sessions three times a week for 10 weeks. The other two schools will act as the controls and continue with their normal routine.

The primary outcomes measures of the study are waist circumference; glucose levels; lipid profiles; blood pressure; 20 m shuttle run test performance; body composition; daily moderate to vigorous physical activity, high-sensitivity C-reactive protein (CRP) levels and carotid- artery intima-media thickness (recognised risk factors for cardiovascular disease). The lipid profiles, glucose and CRP will be measured through a simple clinical point-of-care system, using finger prick whole blood automated analysis (Cholestech, LDX). Carotid-artery intima-media thickness will be assessed using B-mode ultrasound scanning. Body composition will be measured through octapolar impedance (InBody 720), 20 m shuttle run test performance through the British National Coaching Foundation protocol Ramsbottom et al., 1988), and waist circumference via an anthropometric tape measure (Gulick). Blood pressure will be determined using the Omron M5-I blood pressure monitor and total physical activity through uniaxial accelerometry (Actigraph GT1M). Additionally, the participants’ maturity will be assessed through anthropometric measurements using the method described by Mirwald et al., (2002^2^). All measures will be assessed in the control and interventions groups at baseline and at the end of the intervention (10 weeks).

***Participants***

The participants will be 100 Year 9 pupils (aged 13-14 years), drawn from physical education classes at four secondary schools in Northeast England. There will be approximately 50 participants in the intervention arm of the study and 50 in the control; thus each school will have approximately 25 participants. The participants will be given a written and verbal explanation of the procedures and parental and child informed consent and an Exercise and Physical Activity Readiness Assessment for Children and Adolescents form will be provided for all participants.

*Exclusion criteria*

- Condition or injury or co-morbidity affecting the young person’s ability to undertake exercise.
- Symptoms of or known presence of heart disease or major atherosclerotic cardiovascular disease
- Diabetes Mellitus
- Early family history of sudden cardiac death
- Condition or disorder which is communicable via blood
- Pregnancy or likelihood of pregnancy

Participants reporting that they are asthmatic will be allowed to participate with medical clearance from their GP and providing that they use their bronchodilator medication before exercise if recommended. All participants will undergo adequate warm-up and exercise will not be conducted in cold, dry conditions associated with increased risk of bronchospasm.

All pupils from one Year 9 physical education class at four local secondary schools will be invited to participate in the study through a presentation by K Weston. This will be done during a physical education lesson. During this the study will be introduced and K Weston will provide demonstrations of how some of the measurements will be obtained. It is hoped this will aid recruitment and retention.

Participants who are willing to participate, have received parental consent, have returned the Exercise and Physical Activity Readiness Assessment for Children and Adolescents form and are free from any of the exclusion criteria will take part on the study.

***Sample size***

The sample size is a convenience sample of N=100, from the Year 9 school group at four local schools. This study is clearly defined as a pilot/ exploratory trial; therefore, in accordance with the MRC guidance on developing and evaluating complex interventions (Craig et al., 2008), the pilot is intended to inform sample size estimations for a subsequent trial. Performing a 'power calculation' in a pilot study is counterintuitive, as one of the key reasons for doing the pilot is to permit secure assumptions about effect size and variability - the very information needed for a power calculation. A sample size of 100 will allow us to evaluate whether the intervention can be delivered as intended and to examine compliance and retention to inform a future definitive study.

***Assessment of health outcome measures and physical activity***

The measurement of the various health outcome measures will be completed during participants’ physical education lessons. All measures will be assessed before the start of intervention (baseline) and at the end of the intervention (10 weeks).

1. *Lipid profiles, glucose and CRP measurements*

The blood measurements will be assessed through a simple automatic blood analyser (Cholestech, LDX) and uses finger prick whole blood. The researcher taking the blood samples (K Weston) was trained by a member of the Cholestech LDX training team in June 2010. Medical gloves will be worn by the researcher at all times. Participants will sit quietly for 5 minutes before sample collection. After checking that their hand is warm and relaxed, the researcher will clean the participants’ finger tip with an alcohol swab and dry it with a gauze pad. The participants’ hand will be held securely to prevent any sudden movement, and a firm quick puncture will be made on the participant’s middle finger on either hand using a finger prick stick (Unistik 3). The first large drop of blood will be wiped away, and the finger then gently squeezed until another large drop appears. The finger will then be held downward and pressure gently applied to help the blood flow more freely. The researcher will collect the blood using a capillary tube. After the tube has been filled, the participant will be asked to press a gauze pad on the puncture site for a few seconds until the bleeding stops. The blood will be distributed into an analysing cassette which is then inserted into the blood analyser. After 5 minutes the analyser will print the results. This process will be enacted to obtain the lipid profiles and glucose measurements, and then repeated to get the participants’ CRP results. All materials contaminated with blood will be disposed of in a biohazardous waste container/sharps bin.

1. *Blood Pressure*

For five minutes before and during these measurements, participants will be seated. Blood pressure will be determined using the digital automatic Omron M5-1 blood pressure monitor. Measurements will be taken on the right arm and participants will be asked to sit with their arm resting on a table at 90°.

1. *Maturity*

Peak height velocity (a maturity offset value) will be predicted using multiple regression equations which include three somatic dimensions (height, sitting height and leg length). Thus, participants’ height, sitting height and leg length will be measured to the nearest 0.1cm, and two measurements will be taken for each variable.

1. *Body Composition*

Height, weight and body composition will be individually measured and recorded to the nearest 0.1cm by a stadiometer and 0.1kg by seca scales respectively. Height will be measured by a stadiometer and body composition through octopolar impedance (InBody 720). Participants will be asked to remove their shoes and socks and stand on a scale and grip the hand electrodes. The device will then measure their percent body fat and skeletal muscle mass.

1. *20m shuttle run test*

To indirectly assess the participants’ aerobic fitness, the standardised and widely used multistage 20m shuttle run test (Ramsbottom et al., 1988) will be administered. Participants will be asked to wear a heart rate monitor which will record their heart rate responses during the test. They will then run continuously between two cones set 20m apart to the rhythm of sound beeps coming from the test CD. Participants will continue to run until they miss two consecutive beats. The number of runs completed by each participant will be recorded and from this a peak value obtained.

1. *Waist circumference*

Participants will be asked to remove any clothing that restricts easy access to the waist and abdomen and should stand straight with arms at side, feet together and abdomen relaxed. The tape measure (Gulick) will then be placed around the participants’ bare midriff midway between the tenth rib and the iliac crest. The researcher will ensure that the tape is parallel to the floor the whole way round and that the tape is taut but not pressing against the skin. The participant will be asked to breathe out normally and the measurement will be taken at the end of this breath. This will be repeated to ensure accuracy and recorded to the nearest 0.1cm.

1. *Physical Activity Monitoring*

Daily physical activity will be measured using uniaxial accelerometry (Actigraph GT1M). The accelerometer will be worn on a belt around the hip for seven days. Measurements will be taken continually for these seven days.

1. *Carotid-artery intima-media thickness*

This is a non-invasive test which will be performed by a trained sonographer (George) using a high-resolution B-mode ultrasound transducer. The test is safe, painless and takes approximately 15 minutes. Participants will be asked to lie down on a mat with their head facing away from the side of the neck that is going to be scanned first. After applying conducting gel to the skin on the participants’ neck, a small hand-held transducer is applied to image the carotid arteries. Scans will then be taken from three different angles (anterior, lateral and posterior) on each side of the neck.

***Exercise intervention***

The fun short physical activity blasts sessions will be conducted by K Weston who holds a valid CRB Enhanced Disclosure Certificate and have experience in instructing and leading physical activity sessions. The project will run over ten school weeks and will take place three times a week during the school physical education lessons and after school/ during lunch breaks. Participants will have the choice of taking part in either dance activities, non-contact boxing drills and football fitness drills. They will also be able to change activity on a weekly basis, should they wish to do so.

At the start of each session, participants will be briefed and given the opportunity to ask questions.

Each physical activity session will comprise of three distinct sections; a 5 minute warm up, the main fun short physical activity blasts section which will last for approximately 15 minutes and a 5 minute cool down. The warm up will include pulse raising activities relevant to the physical activity mode of the session and stretching. The cool down phase will consist of gentle pulse lowering activities and stretching. In the main part of the session there will be approximately four to seven high-intensity fun short physical activity blasts, which will then be followed by 1 minute and 30 seconds of recovery work at a low intensity. Each high-intensity activity blast will last for 45 seconds, and will include activities such as jumping up and down on the spot, sprinting and then kicking a football and punching boxing focus pads. In line with the literature on high-intensity exercise, the participants should aim to attain a heart rate of approximately 90-95% of their maximum heart rate during the high-intensity short blasts.

The total duration of each session will be approximately 30 minutes. To protect the fidelity of the intervention; and to ensure that the activities are indeed of a high intensity; participants will be asked to wear heart rate monitors (Polar RS400) during their sessions and their heart rate responses will be recorded. This will allow the investigators to monitor the participants’ heart rate responses to the intervention, and where necessary can then adjust the intervention accordingly. During these sessions, the heart rate monitors will be explained to the participants and they will then fit them and keep them on for the remainder of the session.

If any participants wish to withdraw during the sessions the following arrangements will be enacted. The session will be paused and the researcher will thank the participant for their involvement. The participant will then report to their physical education teacher. When the physical activity sessions have been completed, all participants will be thanked for taking part and will be provided with information about how they can access the results of the research.

In order to maximise the impact of the exercise sessions and allow this to be explored fully; it will be explained to intervention group participants that they will be expected to complete as many of the exercise sessions as possible. Thus, to aid adherence to the programme, incentives such as t-shirts and water bottles will be awarded to those who complete at least 70% of the sessions. Those completing 90% of sessions will be entered into a pirze draw to win a pair of training shoes. All participants who complete the baseline and post intervention testing will receive a small thank you pack. All data collected will be anonymised, stored in locked filing cabinets and password protected computers at Teesside University. After the project is completed information will be stored securely for up to six years by Teesside University and then destroyed.

***Data Analysis***

The primary and secondary outcome measures will be analysed using an ANCOVA model to account for chance imbalances at baseline (covariate = baseline value; Dependent variable = change from baseline to follow-up). The general model is (Vickers & Altman, 2001): Change = constant + a×(baseline) + b×(group; 0,1), where ‘group’ is coded ‘0’ for the control arm and ‘1’ for the intervention, and coefficient ‘b’ provides the difference between the mean change scores for each arm (intervention minus control). A 90% confidence interval for this difference will also be calculated and presented.

**References**

Babraj, J.A., Vollaard, N.B.J., Keast, C., Guppy, F.M, Cottrell, G., & Timmons, J.A., 2009. Extremely short duration high intensity interval training substantially improves insulin action in young healthy males. *BMC Endocrine Disorders* [e journal] 9 (3). Available at http://www.biomedcentral.com/1472-6823/9/3 [Accessed on 9th October 2009].

Burgomaster, K.A., Hughes, S.C., Heigenhauser, G. J., Bradwell, S.N., & Gibala M.J., 2005. Six sessions of sprint interval training increases muscle oxidative potential and cycle endurance capacity in humans. *Journal of Applied Physiology,* 98 (6): pp.1985-1990.

Burgomaster, K.A., Heigenhauser, G.J.F., & Gibala, M.J., 2006. Effect of short-term interval training on human skeletal muscle carbohydrate metabolism during exercise and time-trial performance. *Journal of Applied Physiology*, 100: pp.2041-2047.

Burgomaster, K.A., Cermak, N.M., Phillips, S.M., Benton, C.R., Bonen, A., & Gibala, M.J., 2007. Divergent response of metabolite transport proteins in human skeletal muscle after sprint interval training and detraining. *American Journal of Physiology. Regulatory, integrative and comparative physiology*, 292: pp.R1970-R1976.

Burgomaster, K.A. et al., 2008. Similar metabolic adaptations during exercise after low volume sprint volume interval and traditional endurance training in humans. *Journal of Physiology* 586: pp. 151-160.

Chia, M., & Armstrong, N., 2007. Maximal Intensity Exercise. In Neil Armstrong eds. *Advances in Sport and Exercise Science Series: Paediatric Exercise Physiology*. Edinburgh: Churchill Livingstone Elsevier:

Craig, P. et al., 2008. Developing and evaluating complex interventions: the new Medical Research Council guidance. *British Medical Journal*, 337: pp. 1281-1283.

Mirwald, R.L. et al., 2002. An assessment of maturity from anthropometric measurements. *Medicine and Science in Sports and Exercise,* 34 (4): pp. 689-694.

Tjønna, A.E., et al., 2009. Aerobic interval training reduces cardiovascular risk factors more than a multitreatment approach in overweight adolescents. *Clinical Science*, 116: pp. 317-326.

Vickers, A.J. & Altman D.G., 2001. *Analysing controlled trials with baseline and follow up measurements*. British Medical Journal, 323: pp.1123-1124.

Weiss, R. et al., 2004. Obesity and the metabolic syndrome in children and adolescents. *New England Journal of Medicine,* 350: pp. 2362-2374.

Whyte, L.J., Gill, J.M.R., & Cathcart, A.J., 2010. Effect of 2 weeks of sprint interval training on health-related outcomes in sedentary overweight/obese men. *Metabolism Clinical and Experimental*, 59: pp.1421-1428.

Appendix B: Head Teacher’s Consent Form

HEAD TEACHER’S CONSENT FORM FOR SCHOOL PARTICIPATION

**Assessment of various health outcome measures and physical activity levels in Year 9 pupils**

Please read the statements and confirm your consent by putting your initials in the appropriate boxes and complete the information at the bottom if you would like your school to take part in this study:

| 1. I confirm that I have read and understood the information sheet provided in February 2011 and have had the opportunity to ask questions (face to face and by telephone). |  |
| --- | --- |
|  |  |
| 1. I understand that the school’s participation is entirely voluntary and we can discontinue our participation at any time prior to data analysis (22^nd^ July 2011), without giving a reason. |  |
|  |  |
| 1. I understand that all participants’ results will be anonymous and confidential, and that only staff of Teesside University directly involved in the project will have access to the raw data. 2. I confirm that blood samples can to be taken and ultrasound scanning can take place on School premises as described in the information sheet. |  |
|  |
|  |  |
| 1. I understand that parents and children will be asked to provide consent to participate in the study and that the school should not persuade or discourage any pupil to take part. |  |

Name of School: ……………………………………………………………………..

Name of Head Teacher: …………………………………………………………….

Signature of Head Teacher: …………………………………………………………

Date: ………………………………………………………………………………….

If you have any questions please contact Kathryn Weston.

Appendix C: Parental/ Guardian Consent Form (control schools)

# PARENTAL OR GUARDIAN CONSENT FORM FOR CHILD PARTICIPATION

**Assessment of various health outcome measures and physical activity levels in Year 9 pupils**

**Please initial box if you agree**

I have read and understood the information sheet(s) provided in February 2011

for the above study and have had the opportunity to ask questions about the study.

I understand that participation in this study is entirely voluntary and I and/or

my child can withdraw at any time prior to 22^nd^ July 2011 without giving any

reason and without any of my rights/the rights of my child being affected.

I understand that all information will be treated as confidential, and that my

child will not be identified in any way.

I understand that useful health findings may be found from some of the

Measurements taken, and that information will be fed back to me as described

in the information sheet.

I understand that all hard copies of data collected will be stored in a locked

filing cabinet in the office where the lead researcher is based at Teesside

University, and any electronic files will be stored on password protected

computers and all raw data will be destroyed once the study and subsequent

analyses are completed.

I agree that the anonymised data collected about my child during this study may be

held securely (as described in the information sheet) for a minimum of 20 years

and that it may be used for future research if an appropriate ethics committee has

approved that research.

I verify that my child meets the inclusion criteria and is free of all exclusion criteria.

I am happy for my child to be asked if they would like to participate in this project.

---------------------------- __/__/____

Name of Child Child’s date of birth

---------------------------- ---------- -----------------------------

Name of Parent/guardian Date Parent/guardian Signature

Appendix D: Parental/ Guardian Consent Form (intervention schools)

# PARENTAL OR GUARDIAN CONSENT FORM FOR CHILD PARTICIPATION

**Assessment of various health outcome measures and physical activity levels in Year 9 pupils**

**Please initial box if you agree**

I have read and understood the information sheet(s) provided in February 2011

for the above study and have had the opportunity to ask questions about the study.

I understand that participation in this study is entirely voluntary and I and/or

my child can withdraw at any time prior to 22^nd^ July 2011 without giving any

reason and without any of my rights/the rights of my child being affected.

I understand that all information will be treated as confidential, and that my

child will not be identified in any way.

I understand that useful health findings may be found from some of the

Measurements taken, and that information will be fed back to me as described

in the information sheet.

I understand that all hard copies of data collected will be stored in a locked

filing cabinet in the office where the lead researcher is based at Teesside

University, and any electronic files will be stored on password protected

computers and all raw data will be destroyed once the study and subsequent

analyses are completed.

I agree that the anonymised data collected about my child during this study may be

held securely (as described in the information sheet) for a minimum of 20 years

and that it may be used for future research if an appropriate ethics committee has

approved that research.

I verify that my child meets the inclusion criteria and is free of all exclusion criteria.

I am happy for my child to be asked if they would like to participate in this project.

I am happy for my child to be asked if they would like to participate in the physical

activity blasts programme as described in the information sheet; and that they do so

if they wish.

---------------------------- __/__/____

Name of Child Child’s date of birth

---------------------------- ---------- -----------------------------

Name of Parent/guardian Date Parent/guardian Signature

Appendix E: Young person’s Assent Form

# YOUNG PERSON’S ASSENT FORM

**Assessment of various health outcome measures and physical activity levels in Year 9 pupils**

(To be completed by the young person)

Young person to please circle all they agree with:

| Have you read (or had read to you) about this project? | Yes/No |
| --- | --- |
| Has somebody explained this project to you? | Yes/No |
| Do you understand what this project is about? | Yes/No |
| Have you asked all the questions you want? | Yes/No |
| Have you had your questions answered in a way that you understand? | Yes/No |
| Do you understand it is okay to stop taking part at any time? | Yes/No |
| Are you happy to take part? | Yes/No |

If **any** of these answers are ‘no’ or you don’t want to take part, don’t sign your name!

If you **do** want to take part, then you should write your name below:

Your name: Date:

The researcher who explained this project to you needs to sign too:

Print Name: Sign:

Date:

### Please give this form to the researcher OR to your parent/guardian OR to your teacher who will pass this on to us.

Thank you for your help.

Appendix F: Letter to head teacher

Dear Sir/Madam,

I am writing to you to invite your school to participate in a study conducted by researchers at the School of Health and Social Care at Teesside University. The project is entitled ‘Assessment of various health outcome measures and physical activity levels in Year 9 pupils. The attached information sheets provide details on the study.

If you would like to take part in this project, please complete and sign the attached consent form and return it to the address below.

**Please return the signed form within the next seven days in order for you to be eligible to participate.**

Participation is voluntary and your school is free to withdraw from the study without giving a reason before 22^nd^ July 2011. I understand the pressures on your time, but would be very grateful if you were able to support this research. If you have any questions please do not hesitate to contact me using the details below.

Many thanks,

Kathryn Weston

School of Health and Social Care

Teesside University

Middlesbrough, TS1 3BA

**🕿** 01642 342988

**🖂** k.weston@tees.ac.uk

Appendix G: Head teacher information sheet (control schools)

**HEAD TEACHER INFORMATION SHEET**

**Assessment of various health outcome measures and physical activity levels in Year 9 pupils**

**Researchers: Kathryn Weston, Prof. Alan Batterham, Dr. Liane Azevedo, Dr Matthew Weston & Prof. Keith George**

Your school is being invited to take part in a research project. This information sheet will explain what the research is about, the reason it is being done and what your school will be required to do if you decide to take part.

**What does this project aim to do?**

This study aims to asses various health outcome measures and physical activity levels in Year 9 pupils. This will be done by obtaining and examining the following measurements from participants: blood pressure, maturity, finger prick blood profiles, aerobic fitness, body composition, waist circumference, artery thickness and physical activity levels. This information will increase our understanding of these measures in relation to young people.

**Who do we want to work with?**

Approximately 25 children in Year 9 will be invited to participate in the study. It is up to parent/guardian to decide whether or not they want their child to take part. Only children whose parent/guardian have provided consent for them to take part and are willing to participate will be included in the study. In case of discrepancy between parent/guardian consent and child assent the child will be unable to participate in the study.

All parents and/or children have the right to withdraw from the study at any time, without giving a reason up to 22^nd^ July 2011. Finally, children with any of the following conditions will not be able to take part:

- Cold/flu or respiratory infections
- Condition of injury or co-morbidity affecting their ability to undertake exercise.
- Heart or vascular complaints
- Early family history of sudden death
- Condition or disorder which is communicable via blood
- Pregnancy or likelihood of pregnancy

**How will this study run?**

This study will run in your school between 7^th^ February and 22^nd^ July 2011 (dates to be confirmed (TBC)). Pupils who have given their and their parent/guardians informed consent and returned the exercise and physical activity readiness assessment form will be invited to participate in sessions where various health outcome measures will be assessed. These will be conducted by trained researchers from Teesside University and Liverpool John Moores University. All researchers will hold a CRB Enhanced Disclosure Certificate.

The measurements will be taken once in March 2011 and once in July 2011 (dates TBC). They will be collected over visits to the school and will take place during the participants’ physical education lessons. At the start of each session, the study will be explained to the pupils and they will then have the opportunity to ask any questions.

Measurements will be taken of participants’ blood lipid and glucose profiles (via a finger prick sample), carotid artery intima media thickness, blood pressure, maturity, body composition, aerobic fitness, waist circumference and physical activity.

- **Blood samples**: These will be obtained through a quick finger prick and tube collection; and assessed through a simple automatic blood analyser which processes the sample in 5 minutes. Medical gloves will be worn by the researcher at all times and all materials contaminated with blood will be disposed of in a biohazardous waste container/sharps bin.
- **Blood pressure**: Taken using a digital automatic blood pressure monitor.
- **Maturity**: Assessed by measuring participants’ height, sitting height and leg length.
- **Body composition**: Through a device called the InBody which measures percentage body fat, lean body mass and fat body mass.
- **Aerobic fitness**: The participants will be asked to complete a ‘bleep test’, which they may have done at school before. During this, they will be asked to wear a heart rate monitor which will record their heart rate during the test. They will then run continuously between two cones set 20m apart to the rhythm of sound beeps and will keep going until they miss two consecutive beats or they choose to stop.
- **Waist circumference**: Measured by passing a tape measure round the participants’ bare stomach
- **Physical Activity**: Measured through accelerometers will be worn on a belt around the hip for seven days.
- **Carotid-artery thickness**: This is a safe and painless procedure. Participants will be asked to lie down on a mat with their head facing away from the side of the neck that is going to be scanned via ultrasound first. After applying conducting gel to the skin on the participants’ neck, a small hand-held transducer is applied to image the carotid arteries. Scans will then be taken from three different angles on each side of the neck.

If any participants wish to withdraw during the sessions the following arrangements will be enacted. The session will be paused and the researcher will thank the pupil for their involvement. The pupil will then report to their physical education teacher. When the sessions have been completed, all pupils will be thanked for taking part and will be provided with information about how they can access the results of the research. All participants who complete both measurement sessions in February and July will receive a small thank you pack.

**Is the study suitably covered for insurance purposes?**

Through its School of Health and Social Care, Teesside University has agreed to act as sponsor for the proposed study and suitable insurance cover is in place.

**Will the information from this study be kept confidential?**

YES. All participants will be given a unique ID number, which will be used to keep all information collected anonymous and no individual findings will be disclosed to the school. All information collected will remain completely anonymous and will be stored securely in a locked filing cabinet for the length of the project, and/or stored electronically on password protected computers at Teesside University. After the project is completed all the study materials and information will be stored securely by Teesside University for a minimum of 20 years and anonymised data may be used for future study (what is called secondary analysis) but only in research projects that have received ethical approval from an appropriate committee However, should any abusive or unprofessional behaviours or actions be disclosed and/or discovered then confidentiality will be breached and the supervisor will be informed (in the first instance) and if deemed appropriate further actions may be taken.

**What are the possible risks of taking part?**

The multi-stage 20m shuttle run fitness test (commonly known as the ‘bleep test’) is part of the fitness test procedures in all public secondary schools in the UK. In young people, the risks from taking part in high-intensity exercise are very low. Throughout the aerobic fitness test we will monitor the pupils for signs of excessive effort or discomfort. To prevent the risk of heat strain we will ensure that prior to participation the pupils dressed appropriately for exercise and adequately hydrated.

Contamination from the blood measures will be prevented by using one-piece single use finger prick stick and gloves will be worn by the trained researcher at all times. Additionally, all materials contaminated with blood will be disposed of in a biohazardous waste container/sharps bin.

**Who is organising the study?**

The School of Health and Social Care at Teesside University is organising this study.

**What are the benefits of this study?**

The information gained from this study will help to improve our understanding of the various health markers and physical activity in relation to young people. This can then help develop future physical activity programmes for young people.

**What will happen to the results of this study?**

The results will be published in a full report that will be sent to the school. The main findings of the study will be briefed to the school and a summary of this report will be given to all those who took part in the study. We expect this report to be ready by November 2011. Teesside University intends to publish the research findings in an academic journal and they will also be published as part of a doctoral thesis. No reference will be made to individuals in any of the published articles.

**Finally…**

The participation of your school and your pupils is voluntary and you/they may withdraw at any time before 22^nd^ July 2011 (dates to be confirmed) without reason. Your consent for the school to take part does not automatically allow participation of the children. Separate consent will be gained from their parent/ guardian as well as assent from the children themselves.

If you have any further questions, you may contact:

Kathryn Weston

School of Health and Social Care

Teesside University

Middlesbrough, TS1 3BA

**🕿** 01642 342988

**🖂** k.weston@tees.ac.uk

Appendix H:Letter to parents

**Assessment of various health outcome measures and physical activity levels in Year 9 pupils**

Dear Parent/Guardian,

Your child’s school has agreed to take part in a research project with Teesside University. This project is going to examine various health outcome measures and physical activity levels in Year 9 pupils.

Please take time to read the attached information sheet(s) and discuss it with your child.

If you are happy with your child being asked if they would like to take part; and them taking part if they choose to, please complete and sign the attached consent form and the child exercise and physical activity readiness assessment form and return it to your child’s school.

**Please return the signed form within the next two days in order for your child to be eligible to participate.**

Participation is voluntary and your child is free to withdraw from the study at any time before 22^nd^ July 2011 without giving a reason. If you have any questions please do not hesitate to contact Kathryn Weston using the details below.

Many thanks,

Kathryn Weston

School of Health and Social Care

Teesside University

Middlesbrough, TS1 3BA

**🕿** 01642 342988

**🖂** k.weston@tees.ac.uk

Appendix I Parent/Guardian Information sheet (control schools)

**PARENT/GUARDIAN INFORMATION SHEET**

**Assessment of various health outcome measures and physical activity levels in Year 9 pupils**

**Researchers: Kathryn Weston, Prof. Alan Batterham, Dr. Liane Azevedo & Prof. Keith George**

The research team from Teesside University would like to look at various health measures and physical activity levels in Year 9 pupils. This information sheet will help you to decide whether you would like your child to take part in this project. Your child is being invited, along with every other pupil in their physical education class, to take part in this research project.

**What is the purpose of the study?**

This study is going to look at various health outcome measures and physical activity levels in Year 9 pupils. This will be done by collecting the following measurements from participants: blood pressure, maturity, finger prick blood profiles, aerobic fitness, body composition, waist circumference, artery thickness and physical activity levels. This information will increase our understanding of these measures in relation to young people.

**Who will be taking part?**

Approximately 25 pupils in Year 9 will be invited to participate in the study. Only pupils whose parent/guardian have provided consent for them to be asked if they want to take part **and** are willing to participate will be included in the study. In the case of discrepancy between parent/guardian consent and child assent, the child will be unable to participate in the study.

Unfortunately, we can not include any pupils with the following conditions;

- Cold/flu or respiratory infections
- Conditions or injuries that stop your child normally taking part in exercise
- Heart or vascular complaints
- Early family history of sudden death
- Condition or disorder which is communicable via blood
- Pregnancy or likelihood of pregnancy

If your child does not want to take part in the study then they will take part in their normal physical education lesson.

**When will this project run?**

The project will run between 7^th^ February to 22^nd^ July 2011 (dates to be confirmed).

**How will this be done?**

First, if you and your child decide that he/she wants to take part, you must first return the informed consent form, child assent form and the exercise and physical activity readiness assessment form provided. The forms should be returned your child’s PE teacher in the envelope provided to ensure confidentiality. Pupils with informed consent will then be invited to participate in the measurement sessions. These will be conducted by trained researchers from Teesside University who hold CRB Enhanced Disclosure Certificates.

The measurements will be taken once in March 2011 and once in July 2011 (dates TBC). They will be collected over data collection sessions to the school and will take place during the participants’ physical education lessons. At the start of each session, the study will be explained to the pupils and they will then have the opportunity to ask any questions.

A short description of each measurement is detailed below.

- **Blood samples**: These will be obtained by taking a quick finger prick blood sample from your child’s middle finger. The blood will be collected in a small thin tube and assessed through a simple automatic blood analyser which processes the sample in 5 minutes. Medical gloves will be worn by the researcher at all times and all materials contaminated with blood will be disposed of in a biohazardous waste container/sharps bin. No storage of blood will occur.
- **Blood pressure**: Taken using an automatic blood pressure monitor.
- **Maturity**: Assessed by measuring your child’s height, sitting height and leg length.
- **Body composition**: Through a device called the InBody, we will measure your child’s percentage body fat, lean body mass and fat body mass.
- **Aerobic fitness**: Your child will be asked to complete a ‘bleep test’, which they may have done in school before. During this, they will be asked to wear a heart rate monitor which will record their heart rate during the test. They will then run continuously between two cones set 20m apart to the rhythm of sound beeps and will keep going until they miss two consecutive beats or they choose to stop
- **Waist circumference**: We will measure this by passing a tape measure round your child’s bare stomach
- **Physical Activity**: This is measured through physical activity monitors which will be worn on a belt around the hip by your child for seven days. Your child will also record their physical activity in a daily diary during this seven day period. In this they will record the type, duration and intensity of activity they undertake during physical education lessons, school clubs and activities away from school.
- **Carotid-artery thickness**: This is a safe and painless procedure. Your child will be asked to lie down on a mat with their head facing away from the side of the neck that is going to be scanned via ultrasound first. After applying conducting gel to the skin on their neck, a small hand-held transducer is applied to image the carotid arteries. Scans will then be taken from three different angles on each side of the neck.

If your child wishes to withdraw during the sessions the following arrangements will be enacted. The session will be paused and your child will then report to their physical education teacher. When the sessions have been completed, your child will be thanked for taking part and will be provided with information about how they can access the results of the research. All participants who complete both measurement sessions in March and July will receive a small thank you pack.

**Informed consent and confidentiality**

It is up to you to decide whether or not you want your child to take part. To participate in this study it is required that you give your informed consent **and** your child gives their assent. In case of discrepancy between consent and assent your child will be unable to participate in the study. You can withdraw your child from the study at any time up to 22^nd^ July (date to be confirmed) without giving a reason. If you want to withdraw your child you will give us your “child code number” which is provided in the child information sheet, and should be retained and quoted to the researcher (Kathryn Weston, contact information at the bottom of this sheet). If you agree that your child can take part, all the information that we collect from them will be kept strictly confidential, and your child will not be identified in any reports or publications.

**What are the possible benefits of taking part?**

The information gained from this study will help to improve our understanding of the various health markers and physical activity in relation to young people. This can then help develop future physical activity programmes for young people.

**What are the possible risks of taking part?**

The multi-stage 20m shuttle run fitness test (commonly known as the ‘bleep test’) is part of the fitness test procedures in all public secondary schools in the UK. In young people, the risks from taking part in high-intensity exercise are very low. Throughout the aerobic fitness test we will monitor your child for signs of excessive effort or discomfort. To prevent the risk of heat strain we will ensure that prior to participation your child is dressed appropriately for exercise and adequately hydrated.

Contamination from the blood measures will be prevented by using one-piece single used lancets and gloves will be worn by the trained researcher at all times. Additionally, all materials contaminated with blood will be disposed of in a biohazardous waste container/sharps bin.

**Safe storage of information**

All information collected as part of this study will be stored in accordance with the Data Protection Acts (1998). Access to the study materials and data, while the study is underway, will be restricted to members of the research team. Any notes taken and/or any paper based materials you may give us will be stored in a locked filing cabinet for the length of the project, and/or stored electronically on password protected computers at Teesside University. However, if any abusive or unprofessional behaviours or actions are disclosed and/or discovered then confidentiality will be breached and the relevant Head Teachers and/or authorities will be informed. After the project is completed all the study materials and information will be stored securely for a minimum of 20 years and then destroyed.

##### Who will see this information?

The completed report will be sent to your child’s school and a summary of this report will be given to all children who took part in the study. We expect this report to be ready by November 2011. Teesside University intends to publish the research findings in an academic journal and they will also be published as part of a doctoral thesis. Your child will not be identified in any report or journal article.

**Thank you for reading through this information.**

If you have any further questions, please feel free to contact Kathryn Weston:

**🕿** 01642 342934

**🖂** k.weston@tees.ac.uk

Appendix J: Young person information sheet

Young person code number will be

inserted here

**YOUNG PERSON INFORMATION SHEET**

**Assessment of various health outcome measures and physical activity levels in Year 9 pupils**

**Researchers: Kathryn Weston, Prof. Alan Batterham, Dr. Liane Azevedo & Prof. Keith George**

**Why have I been asked to take part?**

You are being asked to take part because we would like to look at various health measurements and physical activity levels of Year 9 pupils. This will help us learn more about young peoples’ health. Unfortunately, if you have any conditions or injuries that stop you normally taking part in exercise then you won’t be able to take part.

**Did anyone else check the study is OK to do?**

Before any research is allowed to happen, it has to be checked by a group of people called a Research Ethics Committee. They make sure that the research is fair. This study has already been checked by Teesside University, School of Health and Social Care Research Governance and Ethics Committee.

**Do I have to take part?**

No you do not have to take part, it is entirely up to you if you want to or not. Also if you decide to take part and then change your mind at anytime before the 22^nd^July 2011 (date to be confirmed) this is fine. If you decide not to take part then you will continue doing your PE lessons as normal.

**What will happen to me if I take part in the research?**

Before you decide if you would like to take part or not we would like you to read the information below carefully and talk about it with your family and friends if you want to. This part tells you about the things we would like you to do. If you and your parent/guardian would like you to take part then you must bring in your signed parent/guardian consent form, your assent form and the form that says if you are ok to take part in physical activity. Without these, you are unable to take part.

You will be invited to take part in measurement sessions which will be held during your PE lesson. The sessions will be taken by a trained researcher from Teesside University. Different things will be measured in each session. Below is some information about each of the tests that we will be doing. Before each session you will have a chance to ask any questions.

For the blood samples we’ll take these by a quick finger prick, and then your sample will be processed in 5 minutes. We’ll record your height, and then your body composition using a machine the InBody which measures percentage body fat, lean body mass and fat body mass. We’ll also measure the size of your waist using a tape measure.

To measure your fitness, we will ask you to do a ‘bleep test’, which you may have done at school before. During this, we will ask you to wear a heart rate monitor which will record your heart rate during the test. You will be asked to run continuously between two cones set 20m apart to the rhythm of sound beeps and to keep going until you miss two beeps in a row.

We will measure your physical activity through a physical activity monitor which will be worn on a belt around your hip for seven days except when showering and sleeping. We will also ask you to record your physical activity in a daily diary during these seven days.

Lastly, we are doing look at an artery in your neck using a ultra sound machine which is completely painless. We will ask you to lie down on a mat and some gel will be applied to the side of your neck. A machine will then create an image of the artery in your neck and we will measure its thickness.

If you wish to withdraw during the session, we will take a quick break and you will report to your PE teacher. When the sessions have finished, we will thank you for taking part and will let you know how you can find out about the results of the research. Everyone who does both measurement sessions in March and July will receive a small thank you pack which will contain a certificate, a sports magazine and a sweat band.

**Might anything about the research upset me?**

We are hoping that nothing we do will be upsetting for you but if at any point you would like to talk about anything that is bothering you, you can tell a member of the project staff, the researcher, your teacher, school nurse or your parents who will be happy to help you with any problems that you may have.

**Will joining in help me?**

We hope that you will enjoy taking part and find the measurement sessions interesting but we cannot promise that taking part will help you. The information we help us learn more about young peoples’ health.

**What will happen when the research stops?**

After the study has been fully completed the information will be kept by Teesside University for a period of six years. It will then be destroyed. The results from the study will be published in an academic journal and as also part of a doctoral thesis. You will not be able to be identified in any report or journal article.

**Will my details and the information I give be kept private if I take part? Will anyone else know I am doing this?**

All the information that you give us will be kept private and will not be shown to your parents, teachers or your friends. You can tell others that you are taking part if you want to. You can withdraw from further participation in the measurement sessions at any time. The only time we would discuss your taking part is if you had a problem and would like to talk about it with someone. Don’t forget you can ask any of the project and research staff questions about the project at anytime as well as your family.

**What do I do next if I would like to take part?**

If you would like to take part you can let your parents/guardian know who will then fill in a form with you that tells us you want to be involved. You and your parent or guardian can then return the forms to your school.

If you have any questions then please ask Kathryn Weston:

**🕿** 01642 342934

**🖂** k.weston@tees.ac.uk

***Thank you!***

Appendix K: Head teacher information sheet (intervention schools only)

**HEAD TEACHER INFORMATION SHEET**

**Assessment of various health outcome measures and physical activity levels in Year 9 pupils**

**Researchers: Kathryn Weston, Prof. Alan Batterham, Dr. Liane Azevedo & Prof. Keith George**

Your school is being invited to take part in a research project. This information sheet will explain what the research is about, the reason it is being done and what your school will be required to do if you decide to take part.

**What does this project aim to do?**

This study aims to asses various health outcome measures and physical activity levels in Year 9 pupils. This will be done by obtaining and examining the following measurements from participants: blood pressure, maturity, finger prick blood profiles, aerobic fitness, body composition, waist circumference, artery thickness and physical activity levels. This information will increase our understanding of these measures in relation to young people.

In addition to the collection of the various health and physical activity measures, your school is also being invited to participate in a 10 week physical activity bursts programme. This programme will run in your school between 7^th^ February and 22^nd^ June 2011 (dates to be confirmed). Pupils who have given their and their parent/guardians informed consent; returned the exercise and physical activity readiness assessment form AND complete the health and physical activity measurement sessions will be invited to participate in these fun short physical activity blasts sessions.

**Who do we want to work with?**

Approximately 25 children in Year 9 will be invited to participate in the study. It is up to parent/guardian to decide whether or not they want their child to take part. Only children whose parent/guardian have provided consent for them to take part and are willing to participate will be included in the study. In case of discrepancy between parent/guardian consent and child assent the child will be unable to participate in the study.

All parents and/or children have the right to withdraw from the study at any time, without giving a reason up to 22^nd^ July 2011. Finally, children with any of the following conditions will not be able to take part:

- Cold/flu or respiratory infections
- Condition of injury or co-morbidity affecting their ability to undertake exercise.
- Heart or vascular complaints
- Early family history of sudden death
- Condition or disorder which is communicable via blood
- Pregnancy or likelihood of pregnancy

**How will this study run?**

This study will run in your school between 7^th^ February and 22^nd^ July 2011 (dates to be confirmed (TBC)). Pupils who have given their and their parent/guardians informed consent and returned the exercise and physical activity readiness assessment form will be invited to participate in sessions where various health outcome measures will be assessed. These will be conducted by trained researchers from Teesside University and Liverpool John Moores University. All researchers will hold a CRB Enhanced Disclosure Certificate. The study will run in two parts; the health measurement sessions and the physical activity sessions. Information about these two parts is detailed below.

***Health markers and physical activity measurement sessions***

The measurements will be taken once in March 2011 and once in June 2011 (dates TBC). At the start of each session, the study will be explained to the pupils and they will then have the opportunity to ask any questions. A short description of each measurement is detailed below.

- **Blood samples**: These will be obtained through a quick finger prick and tube collection; and assessed through a simple automatic blood analyser which processes the sample in 5 minutes. Medical gloves will be worn by the researcher at all times and all materials contaminated with blood will be disposed of in a biohazardous waste container/sharps bin.
- **Blood pressure**: Taken using a digital automatic blood pressure monitor.
- **Maturity**: Assessed by measuring participants’ height, sitting height and leg length.
- **Body composition**: Through a device called the InBody which measures percentage body fat, lean body mass and fat body mass.
- **Aerobic fitness**: The participants will be asked to complete a ‘bleep test’, which they may have done at school before. During this, they will be asked to wear a heart rate monitor which will record their heart rate during the test. They will then run continuously between two cones set 20m apart to the rhythm of sound beeps and will keep going until they miss two consecutive beats or they choose to stop.
- **Waist circumference**: Measured by passing a tape measure round the participants’ bare stomach
- **Physical Activity**: Measured through accelerometers will be worn on a belt around the hip for seven days. Participants will also record their physical activity in a daily diary during this seven day period. In this participants will record the type, duration and intensity of activity they undertake during physical education lessons, school clubs and activities away from school.
- **Carotid-artery thickness**: This is a safe and painless procedure. Participants will be asked to lie down on a mat with their head facing away from the side of the neck that is going to be scanned via ultrasound first. After applying conducting gel to the skin on the participants’ neck, a small hand-held transducer is applied to image the carotid arteries. Scans will then be taken from three different angles on each side of the neck.

***Physical Activity Blasts sessions***

These sessions will run over ten school weeks from March 2011 to June 2011, and will take place three times a week during the school physical education lessons and after school. Participants will have the choice of taking part in either dance activities, non-contact boxing drills and games or football fitness drills. They will also be able to change activity on a weekly basis, should they wish to do so. Pupils taking part in the study will be separated from those who are not. Non-participants will continue with their normal PE lessons in a separate area from the participants during the sessions done in PE time. For the after school sessions, participants will be asked to meet in the school sports hall. The sessions will be conducted by researchers who hold valid CRB Enhanced Disclosure Certificates and have experience in instructing and leading physical activity sessions.

The physical activity sessions will last approximately 30 minutes. The pupils will be asked to wear their indoor PE kit and to bring a bottle of water with them to ensure adequate hydration. At the start of each session, participants will be briefed and given the opportunity to ask questions The sessions will be split into three sections; a 10 minute warm up, the main fun short physical activity blasts and a 5 minute cool down. The warm up will include pulse raising activities relevant to the physical activity mode of the session and stretching. The cool down phase will consist of gentle pulse lowering activities and stretching. In the main part of the session there will be approximately six high-intensity fun short physical activity blasts, which will then be followed by a recovery period.

The fun short physical activity blasts will comprise of 30-60 seconds of high-intensity physical activity, which are designed to increase the pupils’ heart rates to approximately 90% of their maximum. For a 13 year old this would be around 186 beats per minute. Research has shown that this type of exercise better resembles the activity patterns of adolescents, compared with longer activity bouts. The blasts will include activities such as jumping up and down to music, punching boxing focus pads, and sprinting then kicking a football. Each blast will be followed by 1 minute 30 seconds recovery of low intensity activity such as walking on the spot.

Once a week, the participants will be asked to wear heart rate monitors during their sessions and their heart rate will be recorded. This will be done to make sure that they are doing high intensity exercise, which we will be able to tell from their heart rate. During these sessions, the heart rate monitors will be explained to the participants and they will them fit them and keep them on for the remainder of the session. Participants will also be asked to rate how tired they feel using a picture scale.

If any participants wish to withdraw during any of sessions the following arrangements will be enacted. The session will be paused and the researcher will thank the pupil for their involvement. The pupil will then report to their physical education teacher. When the physical activity sessions have been completed, all pupils will be thanked for taking part and will be provided with information about how they can access the results of the research.

To help us look at the impact the programme properly; participants will be expected to complete as many of the exercise sessions as possible. We will therefore offer incentives such as t-shirts and water bottle to those who complete at least 70% of the sessions. All participants who complete the health and physical activity measures testing in both March and July and the exercise sessions will receive a small thank you pack which will contain a certificate, a sports magazine and a sweatband.

**Is the study suitably covered for insurance purposes?**

Through its School of Health and Social Care, Teesside University has agreed to act as sponsor for the proposed study and suitable insurance cover is in place.

**Will the information from this study be kept confidential?**

YES. All participants will be given a unique ID number, which will be used to keep all information collected anonymous and no individual findings will be disclosed to the school. All information collected will remain completely anonymous and will be stored securely in a locked filing cabinet for the length of the project, and/or stored electronically on password protected computers at Teesside University. After the project is completed all the study materials and information will be stored securely by Teesside University for a minimum of 20 years and anonymised data may be used for future study (what is called secondary analysis) but only in research projects that have received ethical approval from an appropriate committee. However, should any abusive or unprofessional behaviours or actions be disclosed and/or discovered then confidentiality will be breached and the supervisor will be informed (in the first instance) and if deemed appropriate further actions may be taken.

**What are the possible risks of taking part?**

The multi-stage 20m shuttle run fitness test (commonly known as the ‘bleep test’) is part of the fitness test procedures in all public secondary schools in the UK. In young people, the risks from taking part in high-intensity exercise are very low. Throughout the aerobic fitness test we will monitor the pupils for signs of excessive effort or discomfort. To prevent the risk of heat strain we will ensure that prior to participation the pupils dressed appropriately for exercise and adequately hydrated. Contamination from the blood measures will be prevented by using one-piece single use finger prick stick and gloves will be worn by the trained researcher at all times. Additionally, all materials contaminated with blood will be disposed of in a biohazardous waste container/sharps bin.

**Who is organising the study?**

The School of Health and Social Care at Teesside University is organising this study.

**What are the benefits of this study?**

The information gained from this study will help to improve our understanding of the various health markers and physical activity in relation to young people. This can then help develop future physical activity programmes for young people.

**What will happen to the results of this study?**

The results will be published in a full report that will be sent to the school. The main findings of the study will be briefed to the school and a summary of this report will be given to all those who took part in the study. We expect this report to be ready by November 2011. Teesside University intends to publish the research findings in an academic journal and they will also be published as part of a doctoral thesis. No reference will be made to individuals in any of the published articles.

**Finally…**

The participation of your school and your pupils is voluntary and you/they may withdraw at any time before 22^nd^ July 2011 (dates to be confirmed) without reason. Your consent for the school to take part does not automatically allow participation of the children. Separate consent will be gained from their parent/ guardian as well as assent from the children themselves.

If you have any further questions, you may contact:

Kathryn Weston

School of Health and Social Care

Teesside University

Middlesbrough, TS1 3BA

**🕿** 01642 342934

**🖂** k.weston@tees.ac.uk

Appendix L: Parent information sheet (intervention schools)

**PARENT/GUARDIAN INFORMATION SHEET**

**Assessment of various health outcome measures and physical activity levels in Year 9 pupils**

**Researchers: Kathryn Weston, Prof. Alan Batterham, Dr. Liane Azevedo & Prof. Keith George**

The research team from Teesside University would like to look at various health measures and physical activity levels in Year 9 pupils. This information sheet will help you to decide whether you would like your child to take part in this project. Your child is being invited, along with every other pupil in their physical education class, to take part.

**What is the purpose of the study?**

This study is going to look at various health outcome measures and physical activity levels in Year 9 pupils. This will be done by collecting the following measurements from participants: blood pressure, maturity, finger prick blood profiles, aerobic fitness, body composition, waist circumference, artery thickness and physical activity levels. This information will increase our understanding of these measures in young people.

As well as the collection of the various health and physical activity measures, your child is also being invited to participate in a 10 week physical activity bursts programme. This study will run in your child’s school between 7^th^ February and 22^nd^ July 2011 (dates to be confirmed). If your child would like to take part they must return your informed consent form, their assent form, the exercise and physical activity readiness assessment form **AND** have completed the health and physical activity measurement sessions. They will then be invited to participate in the fun short physical activity blasts sessions.

**Who will be taking part?**

Approximately 25 pupils in Year 9 will be invited to participate in the study. Only pupils whose parent/guardian have provided consent for them to be asked if they want to take part **and** are willing to participate will be included in the study. In the case of discrepancy between parent/guardian consent and child assent, the child will be unable to participate in the study.

Unfortunately, we can not include any pupils with the following conditions;

- Cold/flu or respiratory infections
- Conditions or injuries that stop your child normally taking part in exercise
- Heart or vascular complaints
- Early family history of sudden death
- Condition or disorder which is communicable via blood
- Pregnancy or likelihood of pregnancy

If your child does not want to take part in the study then they will take part in their normal physical education lesson.

**When will this project run?**

The project will run between 7^th^ February to 22^nd^ July 2011 (dates to be confirmed).

**How will this be done?**

First, if you and your child decide that he/she wants to take part, you must first return the informed consent form, child assent form and the exercise and physical activity readiness assessment form provided. The forms should be returned your child’s PE teacher in the envelope provided to ensure confidentiality Pupils who have returned **all three forms** will then be invited to participate in the measurement sessions. These will be taken by trained researchers from Teesside University and Liverpool John Moores University All researchers will hold a CRB Enhanced Disclosure Certificate. The study will run in two parts; the health measurement sessions and the physical activity sessions. Information about these two parts is detailed below.

***Health markers and physical activity measurement sessions***

The measurements will be taken once in March 2011 and once in July 2011 (dates TBC). They will be collected over data collection visits to the school and will take place during the participants’ physical education lessons. At the start of each session, the study will be explained to the pupils, they will be briefed on how to wear all of the necessary equipment and will then have the opportunity to ask any questions.

A short description of each measurement is detailed below.

- **Blood samples**: These will be obtained by taking a quick finger prick blood sample from your child’s middle finger. The blood will be collected in a small thin tube and assessed through a simple automatic blood analyser which processes the sample in 5 minutes. Medical gloves will be worn by the researcher at all times and all materials contaminated with blood will be disposed of in a biohazardous waste container/sharps bin. No storage of blood will occur. These tests can reveal useful health information and the results will be fed back to you and your child via their test results sheet. Should you wish to discuss any of their results in more detail, please contact Kathryn Weston
- **Blood pressure**: Taken using an automatic blood pressure monitor.
- **Maturity**: Assessed by measuring your child’s height, sitting height and leg length.
- **Body composition**: Through a device called the InBody, we will measure your child’s percentage body fat, lean body mass and fat body mass.
- **Aerobic fitness**: Your child will be asked to complete a ‘bleep test’, which they may have done in school before. During this, they will be asked to wear a heart rate monitor which will record their heart rate during the test. They will then run continuously between two cones set 20m apart to the rhythm of sound beeps and will keep going until they miss two consecutive beats or they choose to stop
- **Waist circumference**: We will measure this by passing a tape measure round your child’s bare stomach
- **Physical Activity**: This is measured through physical activity monitors which will be worn on a belt around the hip by your child for seven days, except when showering, swimming or sleeping. Your child will also record their physical activity in a daily diary during this seven day period. In this they will record the type, duration and intensity of activity they undertake during physical education lessons, school clubs and activities away from school.
- **Carotid-artery thickness**: This is a safe and painless procedure. Your child will be asked to lie down on a mat with their head facing away from the side of the neck that is going to be scanned via ultrasound first. After applying conducting gel to the skin on their neck, a small hand-held transducer is applied to image the carotid arteries. Scans will then be taken from three different angles on each side of the neck. This test can reveal useful health information and the results will be fed back to you and your child via their test results sheet. Should you wish to discuss any of their results in more detail, please contact Kathryn Weston.

***Physical Activity Blasts Sessions***

The project will run over ten school weeks and will take place three times a week during the school physical education lessons and after school. During PE time, pupils who not taking part in the study will continue with their normal PE lessons in a separate area from where the activity blasts sessions are taking place. For the after school sessions, your child will be asked to meet with the other participants in the school sports hall where the session will take place. Your child will have the choice of taking part in either dance activities, non-contact boxing drills and games or football fitness drills. They will also be able to change activity on a weekly basis, should they wish to do so. Your child will be asked to wear their indoor PE kit to the sessions and to bring a bottle of water along with them. There are three parts to each session; a 10 minute warm up, the main fun short physical activity blasts and a 5 minute cool down.

The fun short physical activity blasts will consist of 30-60 seconds of high-intensity physical activities. These are designed to increase your child’s heart rates to approximately 90% of their maximum which for a 13 year old would be around 186 beats per minute. Research has shown that this type of exercise better resembles the activity patterns of adolescents, compared with longer activity bouts. The blasts will include activities such as jumping up and down to music, punching boxing focus pads, and sprinting then kicking a football. Each blast will be followed by 1 minute 30 seconds recovery of low intensity activity such as walking on the spot.

Once a week, your child will be asked to wear heart rate monitors during their sessions and their heart rate will be recorded. This will be done to make sure that they are doing high intensity exercise, which we will be able to tell from their heart rate. During these sessions, the heart rate monitors will be explained to your child and they will them fit them and keep them on for the remainder of the session. Your child will also be asked to rate how tired they feel using a picture scale.

If your child wishes to withdraw during the sessions the following arrangements will be enacted. The session will be paused and your child will then report to their physical education teacher. When the physical activity sessions have been completed, your child will be thanked for taking part and will be provided with information about how they can access the results of the research.

To help us look at the impact the programme properly; your child will be expected to complete as many of the exercise sessions as possible. We will offer incentives such as t-shirts and water bottle to those who complete at least 70% of the sessions. All participants who complete the health and physical activity measures testing in March and July and the exercise sessions will receive a small thank you pack which will contain a certificate, a sports magazine and a sweat band.

**Informed consent and confidentiality**

It is up to you to decide whether or not you want your child to take part. To participate in this study it is required that you give your informed consent **and** your child gives their assent. In case of discrepancy between consent and assent your child will be unable to participate in the study. You can withdraw your child from the study at any time up to 22^nd^ July (date to be confirmed) without giving a reason. If you want to withdraw your child you will give us your “child code number” which is provided in the child information sheet, and should be retained and quoted to the researcher (Kathryn Weston, contact information at the bottom of this sheet). If you agree that your child can take part, all the information that we collect from them will be kept strictly confidential, and your child will not be identified in any reports or publications.

**What are the possible benefits of taking part?**

The information gained from this study will help to improve our understanding of the various health markers and physical activity in relation to young people. This can then help develop future physical activity programmes for young people.

**What are the possible risks of taking part?**

The multi-stage 20m shuttle run fitness test (commonly known as the ‘bleep test’) is part of the fitness test procedures in all public secondary schools in the UK. In young people, the risks from taking part in high-intensity exercise are very low. Throughout the aerobic fitness test we will monitor your child for signs of excessive effort or discomfort. To prevent the risk of heat strain we will ensure that prior to participation your child is dressed appropriately for exercise and adequately hydrated.

Contamination from the blood measures will be prevented by using one-piece single used lancets and gloves will be worn by the trained researcher at all times. Additionally, all materials contaminated with blood will be disposed of in a biohazardous waste container/sharps bin.

**Safe storage of information**

All information collected as part of this study will be stored in accordance with the Data Protection Acts (1998). Access to the study materials and data, while the study is underway, will be restricted to members of the research team. Any notes taken and/or any paper based materials you may give us will be stored in a locked filing cabinet for the length of the project, and/or stored electronically on password protected computers at Teesside University. However, if any abusive or unprofessional behaviours or actions are disclosed and/or discovered then confidentiality will be breached and the relevant Head Teachers and/or authorities will be informed. After the project is completed all the study materials and information will be stored securely for a minimum of 20 years and then destroyed

##### Who will see this information?

The completed report will be sent to your child’s school and a summary of this report will be given to all children who took part in the study. We expect this report to be ready by November 2011. Teesside University intends to publish the research findings in an academic journal and they will also be published as part of a doctoral thesis. Your child will not be identified in any report or journal article.

**Thank you for reading through this information.**

If you have any further questions, please feel free to contact Kathryn Weston:

**🕿** 01642 342934

**🖂** k.weston@tees.ac.uk

Appendix M: Young person information sheet (intervention schools)

Young person code number will be

inserted here

**YOUNG PERSON INFORMATION SHEET**

**Assessment of various health outcome measures and physical activity levels in Year 9 pupils**

**Researchers: Kathryn Weston, Prof. Alan Batterham, Dr. Liane Azevedo & Prof. Keith George**

**Why have I been asked to take part?**

You are being asked to take part because we would like to look at various health measurements and physical activity levels of Year 9 pupils. This will help us learn more about young peoples’ health. We are also inviting you to take part in a 10 week Physical Activity Blasts programme. This will run in your school between 7^th^ February and 22^nd^ July 2011 (dates to be confirmed) and will take place during your PE lessons and after school.

**Did anyone else check the study is OK to do?**

Before any research is allowed to happen, it has to be checked by a group of people called a Research Ethics Committee. They make sure that the research is fair. This study has already been checked by Teesside University, School of Health and Social Care Research Governance and Ethics Committee.

**Do I have to take part?**

No you do not have to take part, it is entirely up to you if you want to or not. Also if you decide to take part and then change your mind at anytime before the 22^nd^July 2011 (date to be confirmed) this is fine. If you decide not to take part then you will continue doing your PE lessons as normal.

**What will happen to me if I take part in the research?**

Before you decide if you would like to take part or not we would like you to read the information below carefully and talk about it with your family and friends if you want to. This part tells you about the things we would like you to do. If you would like to take part then you must return your parent/guardian consent forms, your consent form and the form that says you are ok to take part in exercise. If you don’t bring back these **three** forms then you are unable to take part.

You will be invited to take part in three measurement sessions which will be held during your PE lesson. The sessions will be taken by a trained researchers from Teesside University and Liverpool John Moores University. Different things will be measured in each session.

Below is some information about each of the tests that we will be doing. Before each session you will have a chance to ask any questions.

***Health measurement sessions***

For the blood samples we’ll take these by a quick finger prick, and then your sample will be processed in 5 minutes. We’ll record your height, and then your body composition using a machine the InBody which measures percentage body fat, lean body mass and fat body mass. For this we’ll ask you to take off your socks and shoes. We’ll also measure the size of your waist using a tape measure.

To measure your fitness, we will ask you to do a ‘bleep test’, which you may have done at school before. During this, we will ask you to wear a heart rate monitor which will record your heart rate during the test. You will be asked to run continuously between two cones set 20m apart to the rhythm of sound beeps and to keep going until you miss two beeps in a row, or you choose to stop.

We will measure your physical activity through a physical activity monitor which will be worn on a belt around your hip for seven days except when showering and sleeping. Lastly, we are doing look at an artery in your neck using a ultra sound machine which is completely painless. We will ask you to lie down on a mat and some gel will be applied to the side of your neck. A machine will then create an image of the artery in your neck and we will measure its thickness.

***Physical Activity Blast sessions***

These activity sessions which will take place during your PE lesson and after school. The sessions will run for 10 weeks and for these we would like you to wear your indoor PE kit and bring a bottle of water along. Before we start each session you will be given the opportunity to ask questions.

You will have the choice of taking part in either dance activities, non-contact boxing drills and games or football fitness drills. You will also be allowed to change which activity you do once a week. For the fun activity blasts, you will be doing a fast activity for 30-60 seconds that will cause your heart rate to increase. The blasts will include activities such as jumping up and down to music, punching boxing focus pads, and sprinting then kicking a football. After each blast you will get a rest before you start the next one.

Once a week, we will ask you to wear heart rate monitors during an activity session which will record your heart rate. We will do this to check that the activities are high intensity, which we will be able to tell from your heart rate. During these sessions, we will explain how to use the heart rate monitors, then you will put them on for the rest of the session. We will also ask you how tired you feel using a picture scale.

To help us look at the impact the programme properly; you will be expected to complete as many of the activity sessions as possible. We will offer incentives such as t-shirts and water bottle to those who complete at least 70% of the sessions.

If you wish to withdraw during the session, we will take a quick break and you will report to your PE teacher. When the sessions have finished, we will thank you for taking part and will let you know how you can find out about the results of the research. Everyone who does both measurement sessions in March and July and completes the activity sessions will receive a small thank you pack which will contain a certificate, a sports magazine and a sweat band.

**Might anything about the research upset me?**

We are hoping that nothing we do will be upsetting for you but if at any point you would like to talk about anything that is bothering you, you can tell a member of the project staff, the researcher, your teacher, school nurse or your parents who will be happy to help you with any problems that you may have.

**Will joining in help me?**

We hope that you will enjoy taking part and find the measurement sessions interesting but we cannot promise that taking part will help you. The information we help us learn more about young peoples’ health.

**What will happen when the research stops?**

After the study has been fully completed the information will be kept by Teesside University for a period of six years. It will then be destroyed. The results from the study will be published in an academic journal and as also part of a doctoral thesis. You will not be able to be identified in any report or journal article.

**Will my details and the information I give be kept private if I take part? Will anyone else know I am doing this?**

All the information that you give us will be kept private and will not be shown to your parents, teachers or your friends. You can tell others that you are taking part if you want to. You can withdraw from further participation in the measurement sessions at any time. The only time we would discuss your taking part is if you had a problem and would like to talk about it with someone. Don’t forget you can ask any of the project and research staff questions about the project at anytime as well as your family.

**What do I do next if I would like to take part?**

If you would like to take part you can let your parents/guardian know who will then fill in a form with you that tells us you want to be involved. You and your parent or guardian can then return the forms to your school.

If you have any questions then please ask Kathryn Weston:

Kathryn Weston

School of Health and Social Care

Teesside University

Middlesbrough, TS1 3BA

**🕿** 01642 342934

**🖂** k.weston@tees.ac.uk

***Thank you!***

Appendix N: Exercise and Physical Activity Readiness Assessment for Children and Adolescents

**EXERCISE AND PHYSICAL ACTIVITY READINESS ASSESSMENT FOR CHILDREN AND ADOLESCENTS**

The purpose of this form is to ensure that we provide every participant with the highest level of care. For most children, physical activity provides an opportunity to have fun and promotes the basis for good health. However there are a small number of children or adolescents who may be at risk when participating in an exercise/ physical activity program. **If you want your child to participate in this study the completion of this questionnaire is mandatory.** The information contained in this form is confidential.

**Your child’s personal details**

Your child’s name:

_____________________________________________________________

Your child’s date of birth (day/month/year): ­­­­­­­­­­­­­

__________________________________________

Is your child male or female? (circle one): **MALE FEMALE**

Has your child ever had any of the following? (tick either ‘yes’ or ‘no’ for each condition)

|  | Yes | No |
| --- | --- | --- |
| An operation |  |  |
| A heart condition |  |  |
| Diabetes |  |  |
| Cystic fibrosis |  |  |
| High blood pressure |  |  |
| High cholesterol |  |  |
| Breathing problems or shortness of breath (e.g. asthma) |  |  |
| Coughing during or after exercise |  |  |
| Epilepsy or seizures/convulsions |  |  |
| Fainting |  |  |
|  | Yes | No |
| Dizzy spells |  |  |
| Increased bleeding tendency or haemophilia |  |  |
| Broken bone |  |  |
| Muscular pain whilst exercising in the last 6 months |  |  |
| Joint pain in the last 6 months |  |  |
| Any allergies |  |  |
| Cerebral palsy |  |  |
| ADHD – attention deficit and hyperactivity disorder |  |  |
| Hypermobility |  |  |
| Intellectual impairment |  |  |

If you ticked ‘yes’ for anything above, please give further details here, including how long ago; what treatment was given, if any; and where any pain was/is:

___________________________________________________________________

___________________________________________________________________

___________________________________________________________________

___________________________________________________________________

If your child is currently taking any medication, please state if there are any side effects:

___________________________________________________________________

___________________________________________________________________

Does your child use an inhaler, puffer ventilator for asthma? (circle one)

**YES NO**

**Physical activity and exercise**

Are you aware of any medical reason/condition that might prevent your child from participating in physical activity or exercise? (circle one) **YES NO**

If yes, please explain: _________________________________________________________

Does your child participate in any organized sports? (circle one)

**YES NO**

If yes, what are they? _________________________________________________________

Is there anything else that we should know about your child?

___________________________________________________________________

___________________________________________________________________

I hereby acknowledge that:

- The information provided above regarding my child’s health is, to the best of my knowledge, correct.
- I will inform you immediately if there are any changes to the information provided above.

Parent/Guardian Signature: ______________________________ Date:_______________

Thank you for completing this form. You have now answered all the questions. Now please return the completed form, with your completed reply form indicating whether or not you would like your child to take part in the research to your child’s school teacher (Name of the teacher to be added later).

**For office use:**

Approved for participation: _______________________________ Date: _______________
