# Supplementary material for: Effect of Novel, School-Based High-Intensity Interval Training (HIT) on Cardiometabolic Health in Adolescents: Project FFAB (Fun Fast Activity Blasts) - An Exploratory Controlled Before-And-After Trial
Source: PLoS One. 2016 Aug 3;11(8):e0159116. doi: 10.1371/journal.pone.0159116 (PMC4972319; doi:10.1371/journal.pone.0159116)
Supplement: S1 Table — (DOCX) [file pone.0159116.s005.docx]

Supplementary Table 1. Baseline comparisons of retained participants (complete cases), and those lost to follow-up (incomplete cases)

| Outcome | Mean (± standard deviation) [number of participants] | |
| --- | --- | --- |
|  | **Complete cases^1^** | **Incomplete cases^2^** |
|  |  |  |
| Weight (kg) | 57.7 ± 12.8 [86] | 54.6 ± 3.8 [5] |
| SMM (kg) | 25.6 ± 5.0 [81] | 22.6 ± 2.6 [5] |
| Body fat (%) | 19.0 ± 9.6 [81] | 20.8 ± 5.5 [5] |
| BMI (kg/m^2^) | 21.6 ± 0.8 [86] | 21.0 ± 3.8 [5] |
| BP (sys) (mmHg) | 120 ± 10 [94] | 114 ± 12 [2] |
| BP (dia) (mmHg) | 70 ± 9 [94] | 77 ± 4 [2] |
| WC (cm) | 73.6 ± 12.1 [86] | 69.6 ± 5.9 [10] |
| TC (mmol/L) | 3.79 ± 0.68 [95] | 3.69 ± 0.41 [3] |
| HDL (mmol/L) | 1.42 ± 0.41 [95] | 1.57 ± 0.77 [3] |
| LDL (mmol/L) | 1.59 ± 0.85 [95] | 1.50 ± 0.63 [3] |
| TG (mmol/L) | 0.96 ± 0.56 [95] | 1.32 ± 0.81 [3] |
| GLU (mmol/L) | 5.37 ± 0.72 [95] | 6.00 ± 1.70 [3] |
| hs-CRP (mg/L) | 0.48 ± 0.30 [51] | 0.98 ± 0.40 [2] |
| 20m SRT (shuttles) | 55 ± 22 [87] | 52 ± 39 [4] |
| Daily MVPA (mins) | 59.1 ± 23.7 [28] | 71.9 ± 30.3 [32] |
| Maturity offset (years) | 0.42 ± 1.18 [90] | 0.68 ± 0.97 [7] |
| ^1^ = baseline and post-intervention data available  ^2^ = baseline data available, post-intervention data missing  SMM = skeletal muscle mass  Body fat (%) = percentage body fat  BMI = body mass index  BP (sys or dia) = blood pressure (systolic or diastolic)  WC = waist circumference  TC = total cholesterol  HDL = High density lipoprotein cholesterol  LDL = Low density lipoprotein cholesterol  TG = triglycerides  GLU = glucose  hsCRP = high-sensitivity C-reactive protein  20m SRT performance= 20m shuttle-run test performance  Daily MVPA = daily moderate-to-vigorous physical activity | | |
